# Embedded Cardiorespiratory Monitoring Using a 77 GHz FMCW Radar: On-Chip Multi-Timescale Tiny 1D CNN and Whole-Node Power Evaluation

**DOI:** 10.3390/s26196325

**Published:** 2026-10-07

**Authors:** Anna Ślesicka

**Affiliations:** Department of Navigation, Faculty of Navigation and Logistics, Polish Air Force University, 08-521 Dęblin, Poland; a.slesicka@law.mil.pl

**Keywords:** energy efficiency, FMCW radar, cardiorespiratory monitoring, artificial intelligence, heart rate, respiration rate

## Abstract

A 77-GHz radar can detect small chest movements induced by cardiac activity and respiration; however, typical systems transmit the acquired signals to an external computer for processing. This limits the development of autonomous and energy-efficient monitoring devices. This study presents a system in which radar signal processing, signal quality assessment, and heart rate (HR) and respiratory rate (RR) estimation are performed directly on the AWR1843 radar device. A Tiny 1D CNN used a 10-s observation window for HR and a 30-s window for RR, producing updated estimates every 1 s. The system was validated with 30 participants aged 18–72 years. The mean absolute error was 2.09 beats/min for HR and 1.05 breaths/min for RR. Compared with the implemented short-window FFT-based spectral estimator, the errors were reduced by 45.3% and 37.5%, respectively. INT8 quantization reduced the model weight size from 19.2 to 4.8 kB, the inference time from 4.6 to 1.9 ms, and the incremental energy per network execution from 2.7 to 0.9 mJ. However, this inference-level improvement did not produce a proportional reduction in whole-node power, which remained approximately 2.38 W because the continuously active radar front end and frame-level signal processing dominated the total power consumption. The system transmits only the estimated parameters and their associated reliability indicators. Consequently, the radar can operate as a standalone measurement node rather than continuously streaming raw data to an external computer.

## 1. Introduction

Physiological monitoring systems intended for use in vehicles, residential buildings, and remote care settings should be capable of operating for extended periods without frequent maintenance. Their practical usefulness therefore depends not only on the accuracy of the estimated parameters but also on power consumption, memory footprint, processor load, and the volume of data transmitted to external devices. This issue is particularly important in continuously operating systems because streaming raw data to a computer or cloud platform may increase both energy consumption and communication interface utilization. Local signal processing can reduce the amount of transmitted data. However, the use of artificial intelligence alone does not ensure energy efficiency. The computational, memory access, and communication costs should therefore be evaluated on the actual target device on which the system is intended to operate [1,2,3].

Heart rate (HR) and respiratory rate (RR) are fundamental parameters describing current cardiorespiratory activity. They are typically measured using electrocardiography, photoplethysmography, or respiratory belts. These methods provide reliable reference measurements but require direct contact between the sensor and the body as well as correct sensor placement. During long-term monitoring, these requirements may reduce user comfort and increase the effort required to operate the system. Contactless methods are particularly useful for residential and in-vehicle monitoring. However, their accuracy may be limited by body motion, variable propagation conditions, and the energy requirements of the sensing device [4].

Frequency-modulated continuous-wave (FMCW) radars provide an alternative to contact sensors. They enable contactless measurement of small displacements of the chest surface associated with breathing and mechanical cardiac activity [5,6,7]. Unlike cameras, radar does not capture optical images depicting a person’s appearance, and its operation is independent of lighting conditions. Measurements can also be acquired through clothing and in conditions in which optical sensors are difficult to use. These properties make radar suitable for use in vehicles, residential spaces, and monitoring systems that do not require continuous physical contact between the sensor and the user.

Chest motion modulates the phase of the signal reflected from the body. Respiration-related displacements generally have a larger amplitude and a lower frequency, whereas cardiac-induced displacements are considerably smaller and more susceptible to interference. The short wavelength at 77 GHz increases phase sensitivity to small displacements, while the wide operating bandwidth supports fine range resolution [6,7]. However, these properties do not eliminate the effects of multipath propagation, variations in radar cross-sections, small body movements, respiratory harmonics, or incorrect range-bin selection [4,8].

A conventional processing pipeline includes transformation to the range domain, selection of the range bin containing the reflection from the chest, phase extraction and unwrapping, band-pass filtering, and frequency estimation. The main advantages of these methods are their interpretability and relatively low computational complexity. In addition to direct spectral peak detection, more advanced parametric estimators have also been applied. Yao et al. [9] presented a maximum-likelihood estimator in which the micromotion signal was modeled as a sum of harmonic components. This approach reduces the effects of spectral leakage and the finite frequency resolution of the discrete Fourier transform. However, it increases the computational cost and makes the result more dependent on the assumed signal model.

The accuracy of conventional methods can also deteriorate because of phase jumps, harmonic interference, changes in the participant’s position, and motion artifacts. Yang et al. [8] applied phase continuity tracking and a reference-free signal quality index (SQI), obtaining mean absolute errors of 1.65 beats/min for HR and 0.79 breaths/min for RR in a driver monitoring system. These results indicate that signal quality assessment should be an integral part of the radar measurement pipeline rather than merely a post-experiment procedure for rejecting unreliable data.

In recent years, neural networks that learn temporal representations directly from radar signals have increasingly been used for vital sign estimation. The investigated approaches include self-supervised pretraining using unlabeled radar signals [10], reconstruction of the weak cardiac component in signals acquired with a low-power radar [11], and reconstruction of electrocardiographic waveforms from FMCW radar data [12]. Kim et al. [13] proposed HeartBeatNet, a lightweight model designed specifically for heart rate estimation using convolutional and dilated convolutional layers. These studies demonstrate that data-driven models can exploit information beyond the location of the dominant spectral peak. However, their primary focus is usually estimation accuracy or signal reconstruction rather than the computational and energy costs of operating the complete radar processing pipeline.

Beyond these applications, machine learning in radar-based physiological sensing has been applied not only to HR and RR estimation and physiological waveform reconstruction, but also to physiological event detection, breathing-pattern classification, infection detection, sleep-stage classification, cardiovascular disease assessment, affective computing, biometric authentication, and signal-quality assessment [14]. Nevertheless, comparatively few studies have investigated the computational and energy costs of operating a complete embedded pipeline for radar-based HR and RR estimation.

Deep learning methods can reduce the dependence of the results on manually designed frequency estimators. However, a small number of model parameters or a short execution time measured on a workstation does not demonstrate that the model can operate directly on a radar system-on-chip. The feasibility of such an implementation also depends on data buffer sizes, the number of operations, memory access patterns, the numerical precision used to represent weights and activations, and the set of operations supported by the target processor [3]. Quantization that enables integer arithmetic can reduce memory requirements and model execution cost [15]. However, these benefits should be verified by measurements performed on the target hardware.

Several studies have also investigated on-device radar data processing. Mehrjouseresht et al. [16] used the internal processor of a commercial FMCW radar for person localization and conventional HR and RR estimation based on the continuous wavelet transform. Their study demonstrates that advanced signal processing can be performed within a radar node. However, it does not include embedded neural network inference or an analysis of the energy consumed during neural network execution.

The study by Khan et al. [17] is more directly related to the present work. The authors proposed two temporal convolutional neural networks (T-CNNs) designed separately for HR and RR estimation. The models were implemented on an external PYNQ-ZU platform and achieved mean absolute errors of 2.37 beats/min for HR and 1.18 breaths/min for RR, with inference times of 12.6 ms and 6.1 ms, respectively. The authors also developed MAVNet to reduce the influence of motion, again using a PYNQ system-on-chip platform [18]. These studies demonstrate that neural network models can be transferred from a workstation to an embedded computing platform. However, the reported systems still require an additional processing device connected to the radar.

To the best of the authors’ knowledge, the reviewed literature does not report a solution that combines phase-based signal processing and neural network inference directly on a 77 GHz radar system-on-chip, simultaneous estimation of HR and RR using their different time scales, measurement of the power consumption of the complete radar node, and a quantitative assessment of the reduction in transmitted data volume. Existing studies also rarely distinguish clearly between the observation window length, result update interval, computation time, and total system latency. Consequently, a system may be described as operating on-device and in real time solely because its inference time is short, even though this time is measured only after the complete input window has been acquired.

An additional difficulty arises from using a single observation window length for both physiological parameters. HR can be estimated from a relatively short signal segment, whereas reliable RR estimation requires the observation of a larger number of respiratory cycles. A window that is too short increases the uncertainty of the RR estimate, while extending a shared window unnecessarily delays HR updates. Accordingly, this study employs a two-timescale Tiny 1D CNN architecture. The HR branch processes a shorter signal segment, whereas the RR branch processes a longer segment that has been downsampled to a lower sampling rate.

To address these limitations, an embedded system for contactless HR and RR monitoring was developed. The system uses the uRAD Automotive HPA v2.0 radar, which operates in the 77 GHz band and is based on the AWR1843 system-on-chip [18,19]. The AWR1843 integrates a radio-frequency transceiver, a C674x digital signal processor, an ARM Cortex-R4F microcontroller, a hardware accelerator for radar processing, and on-chip memory [19]. All stages of the target processing pipeline, including transformation to the range domain, target range-bin selection, phase stabilization, signal quality assessment, input data preparation, and Tiny 1D CNN inference, were implemented in C using the TI mmWave SDK. The external computer is used only for system configuration, reference data acquisition, and result visualization. It does not participate in HR or RR estimation in the intended operating mode.

The main contributions of this study are as follows:

1. An end-to-end processing pipeline in which both radar signal analysis and Tiny 1D CNN inference are performed directly on the AWR1843, without an external processor participating in HR or RR estimation.
2. A two-timescale Tiny 1D CNN architecture. The HR branch analyzes a shorter signal segment, whereas the RR branch processes a longer segment after downsampling. Both branches also receive information about signal quality.
3. An experimental analysis of the relationship between estimation accuracy and energy cost. The analysis includes the average and peak power consumption of the complete radar node, the incremental energy associated with inference, execution time, memory utilization, and stability during continuous operation.
4. A quantitative comparison between the volume of data transmitted during on-device HR and RR estimation and the corresponding volume of raw radar data, together with an assessment of the resulting reduction in communication interface utilization.
5. Subject-independent validation involving 30 participants aged 18 to 72 years. The primary reference measurements were obtained using a Polar H10 sensor and a Vernier GDX-RB respiratory belt, while an ASPEL ECG system was used for supplementary signal verification during selected measurements.

The remainder of this article is organized as follows. Section 2 describes the measurement platform, radar signal processing pipeline, two-timescale Tiny 1D CNN architecture, its implementation on the AWR1843, experimental protocol, and evaluation methods. Section 3 presents the results for HR and RR estimation accuracy under different measurement conditions, quantization effects, execution time, memory utilization, energy consumption, data transmission, and thermal stability. Section 4 discusses the significance of the results, the relationship between accuracy and energy cost, and the limitations of the study. Section 5 summarizes the main conclusions and outlines directions for future research.

## 2. Materials and Methods

### 2.1. Measurement Platform and System Architecture

The measurement setup comprised a radar operating in the 77 GHz band, reference instruments for recording cardiac and respiratory activity, a system for measuring power and energy consumption, equipment for monitoring the module temperature and ambient conditions, and a computer used for system configuration, synchronization, and data acquisition. Heart rate, respiration rate, and the signal quality index were determined directly on the radar device. During target operation, the external computer did not participate in signal processing or neural network inference. Figure 1 presents the measurement setup and the data flow between its main components.

The main component of the measurement setup was a single uRAD Automotive HPA v2.0 radar module (Anteral S.L., Huarte, Spain) based on the AWR1843 device (Texas Instruments Inc., Dallas, TX, USA). According to the manufacturer’s documentation, the module supports operation from 76 to 81 GHz and incorporates three transmit antennas and four receive antennas implemented on the printed circuit board. The declared effective isotropic radiated power, EIRP, is 19 dBm. The nominal field of view is 120° in azimuth and 40° in elevation. The module measures 45 mm × 45 mm × 10 mm and weighs 5 g [20].

The AWR1843 integrates an FMCW transmit and receive front end, analog-to-digital converters, a TI C674x digital signal processor (TI, Dallas, TX, USA), an Arm Cortex-R4F microcontroller (TI, Dallas, TX, USA), and a hardware accelerator for radar signal processing. The device provides a total of 2 MB of on-chip RAM [19]. Its processing and memory resources were used for transformation into the range domain, selection of the range bin containing the reflection from the chest, phase processing, signal quality assessment, input data preparation, and Tiny 1D CNN inference.

The FMCW profile used a start frequency of 77 GHz and a chirp frequency slope of 70 MHz/µs. The chirp ramp duration was 57.14 µs, the idle time between consecutive chirps was 267 µs, and ADC sampling began 7 µs after the start of the ramp. For each chirp, 256 samples were acquired at a sampling rate of 5.209 MS/s. The full chirp ramp bandwidth was 4.00 GHz, whereas the effective bandwidth covered by ADC sampling was 3.44 GHz. This corresponded to a theoretical range resolution of approximately 0.044 m.

The three transmit antennas were activated sequentially using a TDM MIMO scheme. Each frame contained 16 complete MIMO cycles, corresponding to a total of 48 chirps. The frame period was 50 ms, resulting in a frame rate of 20 Hz. Assuming a maximum usable intermediate frequency equal to 0.8 times the ADC sampling rate, the maximum processed range was 8.92 m. The processed range interval extended from 0 to 8.92 m. The maximum unambiguous radial velocity was ±1.00 m/s, and the theoretical velocity resolution was 0.13 m/s. The receiver gain was set to 30 dB.

The radar module was mounted on a Bosch Professional BT 150 tripod (Robert Bosch Power Tools GmbH, Stuttgart, Germany), which provided stable positioning and reproducible adjustment of the antenna height and orientation relative to the participant. The integrated transmit and receive patch antennas were linearly polarized and operated in a co-polarized configuration. The module was mounted with the antenna board in a vertical orientation, and the antenna boresight was directed approximately perpendicular to the anterior chest wall. The nominal azimuth and elevation offsets relative to the center of the participant’s chest were therefore approximately 0°. The center of the antenna aperture was positioned at approximately sternum height. Measurements were performed at distances of 0.5, 1.0, and 1.5 m, depending on the experimental scenario. An unobstructed line of sight was maintained between the radar and the participant. No large metallic objects were located directly within the propagation path. The walls, laboratory furniture, and measurement equipment constituted stationary background reflectors and remained unchanged during each recording.

A Polar H10 sensor (Polar Electro Oy, Kempele, Finland) was used as the primary reference device for heart rate. The sensor recorded a single-channel ECG signal through electrodes integrated into a chest strap and provided raw ECG samples in microvolts at a sampling rate of 130 Hz. Reference HR values were derived from the recorded ECG waveform. The reference respiratory waveform was acquired using a Vernier GDX-RB respiration belt (Vernier Science Education, Beaverton, OR, USA). The device used a force sensor to measure cyclic changes in belt tension caused by respiratory movements of the chest and provided a force signal in newtons at a sampling rate of 10 Hz. In selected measurements, the ECG recordings were additionally verified using an ASPEL AsCARD Grey electrocardiograph (ASPEL S.A., Zabierzów, Poland), which measures electrical potential differences using surface electrodes and records ECG signals at a sampling rate of 1600 Hz per channel. The ASPEL device served a supplementary role and was not used as the primary source of reference values for the complete dataset.

During dataset acquisition, the measurement computer was used to synchronize and record the radar and reference signals. The detailed synchronization procedure is described in Section 2.3. In the target operating mode, the computer received only timestamps, HR and RR values, signal quality indices, and device-status information. It did not perform range-bin selection, phase processing, parameter estimation, or Tiny 1D CNN inference.

The power and energy measurement path, shown in the upper part of Figure 1, comprised a KORAD KA3005P programmable DC power supply (Dongguan Korad Technology Co., Ltd., Dongguan, Guangdong, China), a BXS600 500 Ω, 1.1 A, 640 VA precision shunt resistor (MCP, Jiading District, Shanghai, China), and a RIGOL DS1104B digital oscilloscope (RIGOL Technologies Co., Ltd., Suzhou, China). The radar node was powered through the shunt resistor. The voltage drop across the resistor and the supply voltage were recorded using the oscilloscope. These measurements were used to determine the current drawn by the radar node, instantaneous power, average power, and the energy consumed during the analyzed operating intervals. The surface temperature of the module was recorded using a 5TC-TT-K-30-36 K-type thermocouple (Omega Engineering Inc., Norwalk, CT, USA) connected to a TC-08 thermocouple data logger (Pico Technology Ltd., St Neots, UK). The ambient air temperature and relative humidity were monitored independently using a Testo 608-H1 thermohygrometer (Testo SE & Co. KGaA, Titisee-Neustadt, Germany).

### 2.2. Signal Processing and Embedded Implementation

Radar data processing was performed directly on the AWR1843 device. The processing pipeline included transformation into the range domain, selection of the range bin and receiver channel containing the reflection from the chest, phase extraction and stabilization, preparation of two temporal input representations, and HR and RR estimation using a multi-timescale Tiny 1D CNN. The slow-time phase signal was sampled at 20 Hz, corresponding to the radar frame rate. This slow-time sampling rate was distinct from the ADC sampling rate used to acquire samples within each chirp. Figure 2 presents the complete signal processing pipeline, its division into the HR and RR branches, and the procedure used to prepare and deploy the model.

Each radar frame comprised 48 chirps organized into 16 TDM MIMO cycles involving three sequentially activated transmit antennas. For the cardiorespiratory phase-processing branch, one chirp corresponding to the same fixed transmit antenna and the same position in the TDM sequence was selected from every frame. The remaining chirps were not combined in the phase-extraction branch. Consequently, the selected chirp provided one phase observation per frame, resulting in a slow-time sampling rate of 20 Hz. A range FFT was applied separately to the 256 intermediate-frequency samples acquired by each of the four physical receive channels. In this study, the term receive channel denotes a physical Rx channel rather than a virtual Tx–Rx pair. The resulting range spectrum was expressed as follows:

(1) $$X_{q}\left[k,m\right]=\sum_{n=0}^{N_{s}-1}w\left[n\right]x_{q}\left[n,m\right]exp\left(-j\frac{2\pi kn}{N_{s}}\right),$$

where *x_q_*[*n*,*m*] denotes the *n*-th intermediate-frequency sample acquired by the *q*-th physical receive channel from the selected chirp in the *m*-th frame, *N_s_* is the number of samples per chirp, *w*[*n*] denotes the window function, and *k* is the range-bin index. Before the transform was computed, the mean value was subtracted from the signal. A Hann window was then applied to reduce spectral leakage.

The chest reflection was located using the noncoherent sum of the receive-channel energies:

(2) $$P\left[k,m\right]=\sum_{q=1}^{N_{Rx}}{\left|X_{q}\left[k,m\right]\right|}^{2}.$$

where *N_Rx_* = 4 denotes the number of physical receive channels. The noncoherent summation was used only to determine and track the target range bin. After the target bin had been selected, the physical receive channel with the highest mean SNR was selected during the initial segment and retained throughout the complete recording for phase extraction.

The restricted target-range interval was defined manually for each measurement scenario on the basis of the known nominal distance between the participant and the radar. The target range bin was then selected automatically within this interval. During the initial segment of each measurement, the bin with the highest mean energy was selected. In subsequent frames, the reflection energy, the continuity of the target position, and phase continuity were considered to reduce spurious switching caused by multipath propagation or small changes in the participant’s position. If tracking moved to an adjacent range bin, the candidate whose wrapped phase was most consistent with the preceding phase sample was selected. The resulting slow-time phase sequence was subsequently unwrapped in the temporal direction. The physical receive channel with the highest mean signal-to-noise ratio within the tracked range bin was selected during the initial segment and retained throughout the entire recording. Therefore, no switching between receive channels and no channel-dependent phase correction were required during a recording.

The raw phase of the selected complex-valued signal was calculated as follows:

(3) $$\tilde{\phi }\left[m\right]=atan2\left(Im\left\{X_{q*}\left[k^{*},m\right]\right\},Re\left\{X_{q*}\left[k^{*},m\right]\right\}\right),$$

Here, k* and q* denote the selected range bin and receive channel, respectively. The phase was then unwrapped over time:

(4) $$\phi \left[m\right]=unwrap\left\{\tilde{\phi }\left[m\right]\right\}.$$

The displacement corresponding to the motion of the chest surface was calculated using the phase-to-displacement relationship for a monostatic radar:

(5) $$∆R\left[m\right]=\frac{\lambda }{4\pi }\left(\phi \left[m\right]-\phi \left[m_{0}\right]\right).$$

Here, *λ* is the wavelength, and *ϕ*[*m*_0_] denotes the phase adopted as the reference value. The DC component and slow drift caused by small changes in the participant’s position were removed from the resulting displacement signal.

Phase-continuity tracking and the reference-free Signal Quality Index (SQI) were implemented according to the procedure described in [8]. The index accounted for the consistency of consecutive phase segments and the stability of the tracked range bin and was normalized to the interval from 0 to 1. No fixed SQI threshold was used to reject entire windows. Instead, the SQI value was provided to the model as additional information about signal reliability and included in the results transmitted by the device.

Two inputs representing different timescales were generated from the stabilized displacement signal. The HR branch used a 10 s window containing 200 samples at a sampling rate of 20 Hz. The signal was processed using a causal fourth-order Butterworth band-pass filter with a passband of 0.7–3.5 Hz, corresponding to heart rates of approximately 42–210 beats/min. The filter was implemented in second-order-section form using biquad filters to improve numerical stability in the embedded implementation.

The RR branch processed a 30 s signal window. Before reducing the sampling rate, the signal was processed using a causal fourth-order Butterworth band-pass filter with a passband of 0.1–0.7 Hz, corresponding to respiration rates of 6–42 breaths/min. The filter was likewise implemented in second-order-section form using biquad filters. The filtered signal was then decimated by a factor of four, from 20 to 5 Hz, resulting in an RR-branch input containing 150 samples. Filtering before decimation reduced the risk of aliasing, whereas the lower number of samples reduced the memory requirements and computational cost.

Each input window was normalized independently according to the following:

(6) $$z_{b}\left[i\right]=\frac{s_{b}\left[i\right]-\mu_{b}}{\sigma_{b}+\varepsilon },$$

Here, *s_b_*[*i*] denotes the *i*-th signal sample in branch *b* ∈ {*HR*, *RR*}, while *μ_b_* and *σ_b_* denote the mean and standard deviation calculated for the corresponding window, respectively. The constant *ε* prevented numerical instability when the standard deviation was close to zero. This normalization reduced sensitivity to amplitude variations associated with the radar-to-participant distance, chest orientation, and interparticipant differences in chest motion.

The two network branches had the same functional structure but did not share weights. They differed in input length and sampling rate, allowing the temporal characteristics of cardiac and respiratory activity to be modeled independently. The network configuration is presented in Table 1.

Each convolutional layer was followed by batch normalization and a ReLU activation. All convolutional layers used a stride of 1 and same padding, so that the temporal length of the feature maps was preserved. In each depthwise-separable convolution, the depthwise stage used a multiplier of 1 and was followed by a pointwise convolution with a kernel size of 1. No temporal pooling was applied before the final global average pooling layer. During inference, the batch-normalization parameters were folded into the parameters of the preceding convolutional layer. The use of depthwise-separable convolutions reduced the number of operations compared with standard one-dimensional convolutions. Dilation rates of 1, 2, 4, and 8 increased the receptive field without requiring large kernels or a deeper network. For the configuration presented in Table 1, the receptive field comprised 67 samples, corresponding to approximately 3.35 s in the HR branch and 13.4 s in the RR branch. Global average pooling along the temporal dimension eliminated the need for large fully connected layers and helped maintain a small model size.

The network was trained offline, outside the radar platform, using synchronized radar and reference signals. The HR labels were derived from the intervals between consecutive R peaks in the ECG signal, whereas the RR labels were derived from the respiratory cycles recorded using the respiration belt. For each radar input window, the reference value was calculated over the corresponding time interval. Before training, the HR and RR target values were standardized using statistics calculated exclusively from the training set. During inference, the inverse transformation was applied to the network outputs to obtain HR in beats/min and RR in breaths/min.

Each regression branch was trained using the Huber loss with a transition parameter of *δ =* 1.0, applied to the standardized target values. For residuals with an absolute value below *δ*, the loss is quadratic, whereas for larger residuals it becomes linear. Compared with the mean squared error, the linear region limits the contribution of individual windows producing large residuals, such as windows contaminated by motion artifacts, and prevents them from dominating the parameter updates. Compared with the mean absolute error, the quadratic region provides smoother gradients near zero and supports stable optimization for typical small estimation errors. Therefore, the Huber loss does not directly suppress motion artifacts in the input signal; rather, it reduces their influence on the training process when they produce outlying residuals.

Each regression branch was optimized using the Adam optimizer with an initial learning rate of 10^−3^ and a mini-batch size of 64. Training was conducted for 20 epochs. After each epoch, the Huber loss was evaluated on the internal validation set, and the checkpoint producing the lowest validation loss was retained as the final floating-point model. A patience-based early-stopping rule was not used.

After the initial floating-point training stage, quantization-aware fine-tuning was performed using the same participant-level training and validation partitions. During this stage, the effects of quantization were simulated for both network weights and intermediate activations. The deployed model used INT8 weights and activations, with weight quantization performed separately for each output channel. Convolution products were accumulated using INT32 arithmetic before requantization to INT8. Before deployment, the batch-normalization parameters were folded into the parameters of the preceding convolutional layers. The final regression outputs were dequantized before the inverse target-standardization transformation was applied to obtain HR in beats/min and RR in breaths/min.

The on-device INT8 implementation was verified using the same set of saved input windows for offline inference and inference on the AWR1843. The corresponding dequantized HR and RR predictions were compared sample by sample. This verification confirmed agreement between the offline and embedded implementations, with the observed numerical differences limited to quantization-rounding effects.

The transformation into the range domain was performed using the AWR1843 hardware accelerator. Phase extraction, phase-continuity tracking, filtering, circular-buffer management, and Tiny 1D CNN inference were executed on the C674x digital signal processor. The ARM Cortex-R4F microcontroller was responsible for radar configuration, data-flow control, execution-time monitoring, and communication with the host computer. The network weights and working buffers were stored in the on-chip memory. Consequently, inference required neither access to external memory nor the transmission of intermediate radar data to the host computer.

The HR and RR buffers were updated upon receipt of each new radar frame, whereas inference was executed once per second. The first HR estimate became available after 10 s of data had been collected, and the first RR estimate became available after the 30 s buffer had been filled. Subsequent estimates were generated at 1 s intervals using overlapping sliding windows.

The output packet transmitted by the device contained a timestamp, the HR and RR estimates, their corresponding signal quality indices, and diagnostic information describing the system status. Raw ADC samples, range profiles, and phase time series were not transmitted during normal operation. Their recording was enabled only in the dataset-acquisition and diagnostic modes. This separation between the data-acquisition mode and the target operating mode enabled the complete estimation pipeline to be executed directly on the radar node and allowed the reduction in communication-interface load to be quantified.

### 2.3. Experimental Protocol and Evaluation Methods

The study involved 30 adult volunteers aged 18–72 years. Before the measurements, the participants were informed about the purpose and procedure of the experiment and provided written informed consent to participate in the study and for the processing of the recorded data. During the measurements, each participant remained seated and faced the radar. The height of the tripod-mounted module was adjusted to align the radar with the central region of the participant’s chest. Measurements were performed through ordinary clothing without any special preparation of the body surface. An example of the participant’s position relative to the radar is shown in Figure 3.

The Polar H10 served as the primary reference device for heart rate, whereas the Vernier GDX-RB belt was used to record the respiratory waveform. The ASPEL AsCARD Grey electrocardiograph was used for supplementary verification of the ECG signal in selected measurements and was not used to generate, combine, or average the reference labels. The radar and reference data were recorded on the same computer using timestamps referenced to a common system clock. At the beginning of each session, the participant performed three slow, deep breaths that were clearly visible in both the radar displacement signal and the respiration-belt waveform. This calibration sequence was excluded from the accuracy analysis and used to verify synchronization. For this purpose, the Vernier waveform was linearly interpolated onto the 20 Hz radar time grid, and a single residual time offset was estimated by locating the maximum cross-correlation between the filtered radar and respiratory signals. The resulting offset was applied to the entire recording.

Each session comprised resting measurements, measurements at different distances from the radar, post-exercise recovery, and controlled motion artifacts. The order of the distances and motion types was varied across participants to reduce potential order effects. The experimental conditions are summarized in Table 2.

In the resting scenario, the participant sat comfortably, breathed naturally, and avoided deliberate movements. During the distance-variation scenario, the tripod position was adjusted relative to the participant without changing the antenna height or orientation. The first 30 s after each change in position were reserved for signal stabilization. This interval and all estimates obtained from windows that overlapped it were excluded from the accuracy analysis.

Before the post-exercise measurement, each participant performed 3 min of moderate-intensity exercise on an elliptical trainer. The resistance level and cadence were adjusted individually to the participant’s physical capacity. The exercise could be terminated at the participant’s request or if any discomfort was reported. Immediately after completing the exercise, the participant assumed the seated measurement position in front of the radar. The recording covered the period of elevated HR and RR and their gradual return toward resting values. The exercise procedure is illustrated in Figure 4.

The controlled-motion recording lasted 180 s per participant and comprised three 30-s periods of instructed movement alternating with three 30-s periods of stillness. The movements included head turns, isolated arm movements, and changes in torso posture while the participant remained seated. Individual movements were performed in response to instructions from the experimenter. These segments were used to evaluate the estimation error and examine the relationship between SQI and estimation quality. The motion intervals were independently annotated in the reference recordings and were not automatically excluded from the test set.

Reference HR labels were calculated from R peaks within the 10 s interval ending at each output time, and reference RR labels were calculated from respiratory cycles within the corresponding 30 s interval. These label durations were retained in the ablation experiments. In particular, the shared-duration model used a 30 s radar input for HR but was evaluated against the same 10 s HR reference label as the nominal model. Consecutive output times were separated by 1 s. For each radar observation window, the reference values were calculated using physiological events occurring within the same temporal boundaries. The reference HR value was calculated from the median of the intervals between consecutive *R* peaks occurring within the analyzed window:

(7) $${HR}_{ref}=\frac{60}{median\left(∆t_{R}\right)},$$

Here, Δ*t_R_* denotes the interval between consecutive *R* peaks, expressed in seconds. The reference *RR* value was determined analogously from the intervals between consecutive respiratory cycles recorded using the Vernier GDX-RB:

(8) $${RR}_{ref}=\frac{60}{median\left(∆t_{B}\right)},$$

Here, Δ*t_B_* denotes the time interval between consecutive respiratory cycles. Because the reference labels were derived from physiological events detected within time-aligned observation windows rather than from a sample-by-sample comparison of the recorded waveforms, resampling to a common frequency was not required for label generation. The automatically detected *R* peaks and respiratory-waveform maxima were visually inspected. Segments containing loss of the reference signal or evident errors from the reference device were excluded before the labels were generated. However, no segments were excluded solely because of poor radar signal quality.

All dataset splits were performed at the participant level before the overlapping windows were generated. Five-fold subject-independent cross-validation was employed. In each fold, data from six participants constituted the test set. Of the remaining 24 participants, 18 were assigned to the training set and six to the internal validation set. The internal validation set was used to select the window lengths, network configuration, best training checkpoint, and quantization parameters. No windows from any test participant were used for training, validation, or hyperparameter selection. The accuracy metrics were calculated from the out-of-fold predictions obtained for participants who were not included in model development in the corresponding cross-validation fold.

The ablation analysis included a comparison of HR windows with lengths of 6, 8, 10, and 12 s and RR windows with lengths of 20, 30, and 40 s. Update periods of 1, 2, and 5 s, as well as three network-size variants, were also examined. The nominal configuration was selected solely on the basis of validation results, taking into account accuracy, memory footprint, and computational cost simultaneously.

Two additional controlled experiments were conducted to assess the effects of the branch-specific observation durations and the SQI input. First, the nominal multi-timescale configuration, using a 10 s HR window and a 30 s RR window, was compared with a shared-duration configuration in which both branches used 30 s observation windows. The 30 s HR input contained 600 samples at 20 Hz, whereas the 30 s RR input contained 150 samples after downsampling to 5 Hz. Apart from the observation duration, the preprocessing procedure, network architecture, training hyperparameters, participant-level data partitions, and evaluation procedure remained unchanged. For the shared-duration comparison, both configurations were evaluated at identical output times from 30 s after initialization onward, using only windows retained for both configurations after reference-signal quality control. The nominal model could also produce HR estimates between 10 and 30 s after initialization, but these earlier estimates were excluded from the paired accuracy comparison.

Second, the contribution of SQI as a network input was evaluated by training an otherwise identical model without SQI. In this variant, the SQI value was not concatenated with the 32-element feature vector produced by global average pooling, and the input dimension of the first fully connected layer was consequently reduced from 33 to 32. All other architectural and training settings were retained. The models used in both additional experiments were trained independently within each cross-validation fold, subjected to the same quantization-aware training procedure, and evaluated on the same participant-independent test sets as the nominal INT8 model.

The floating-point model and the model quantized to INT8 were compared with a classical spectral estimator using the same participant-independent test samples, observation windows, and filtered, preprocessed radar phase signals as the corresponding CNN branches. For HR, 10-s windows sampled at 20 Hz comprised 200 samples and were analyzed using a 200-point FFT. For RR, 30-s windows downsampled to 5 Hz comprised 150 samples and were analyzed using a 150-point FFT. Zero-padding was not applied. After removal of the mean, a Hann window was applied, and the one-sided magnitude spectrum was computed. The FFT bin with the largest spectral magnitude within the predefined physiological HR or RR band was identified, and its frequency was refined using three-point parabolic interpolation based on the dominant bin and its two adjacent bins. The estimated rate was calculated as $60f_{peak}$, where $f_{peak}$ denotes the selected peak frequency in hertz.

Power consumption of the radar node was measured using a RIGOL DS1104B oscilloscope and a precision shunt resistor placed in series in the 5 V power supply path. Two oscilloscope channels recorded the voltages on either side of the shunt relative to a common reference point. The current drawn by the radar was determined as follows:

(9) $$i\left(t\right)=\frac{u_{1}\left(t\right)-u_{2}\left(t\right)}{R_{s}},$$

Here, *u*_1_(*t*) and *u*_2_(*t*) denote the voltages measured before and after the resistor, respectively, and *R_s_* = 0.1 Ω is the nominal shunt resistance. The resistor tolerance was ±0.1%, and the nominal resistance value was used in the current calculation. The instantaneous power drawn by the module was calculated as follows:

(10) $$p\left(t\right)=u_{2}\left(t\right)i\left(t\right).$$

Before the power measurements, the stability of the supply voltage and the compensation of both 10× probes were checked. The probe tips were then connected to the same point to assess the difference between the voltage-channel readings. The observed 2 mV interchannel offset was subtracted when calculating the shunt voltage.

The oscilloscope channels were DC-coupled and connected using passive probes with 10× attenuation. The two supply-voltage channels were set to 1 V/div, and the calculated differential shunt-voltage trace was displayed at 10 mV/div. The GPIO channel was set to 1 V/div. Normal acquisition mode was used with the 20 MHz bandwidth limit enabled and without oscilloscope waveform averaging. Acquisition was triggered by the rising edge of the GPIO signal. For inference-event measurements, the horizontal time base was set to 1 ms/div. The sampling rate was 500 kSa/s, and each 12 ms record contained 6000 samples. For measurements of complete update periods, the horizontal time base was adjusted to cover the interval between successive network invocations.

In the AWR1843 firmware implemented using the Texas Instruments mmWave SDK 3.6, a diagnostic GPIO output was asserted immediately before execution of the HR and RR neural-network branches and deasserted after both inference operations had been completed. The GPIO signal was recorded on the third oscilloscope channel and therefore provided an unambiguous indication of the inference interval Ω. Input preparation and output-message generation were measured separately and were included in the total update time but not in the GPIO-high inference interval.

Per-frame range-domain processing and phase-signal processing were not executed concurrently with neural-network inference. The inference operation was scheduled after completion of the processing associated with the current radar frame. Because the frame period was 50 ms and the complete update required no more than 5.5 ms, the update was completed before processing of the subsequent frame began.

(11) $$E_{Ω}=\int_{Ω}p\left(t\right)dt.$$

The radar acquisition and processing configuration was kept identical during FP32 and INT8 power measurements. The radar remained active between network invocations, and no device sleep mode was enabled between updates.

The reference power *P_ref_* was calculated as the mean power over the complete GPIO-low interval between two successive network invocations. This interval included continuous operation of the radar front end and the routine frame-level processing performed at 20 Hz but excluded the GPIO-high inference interval. The incremental energy attributed to neural-network inference was consequently calculated as follows:

(12) $$E_{inc}=\int_{Ω}[p\left(t\right)-P_{ref}]dt.$$

Thus, *E_inc_* represents the additional energy associated specifically with neural-network inference above the reference power of the continuously operating radar node.

The sensitivity of the incremental-energy estimate to the selected reference power was evaluated by recalculating it for reference values 0.05 W below and above the nominal value of 2.38 W. For the 1.9 ms INT8 inference interval, the resulting energies were 0.995 mJ at 2.33 W, 0.900 mJ at 2.38 W, and 0.805 mJ at 2.43 W.

The mean and peak power of the complete radar node were calculated over the full measurement record and were therefore not restricted to the GPIO-high interval. Measurements were analyzed for update periods of 1, 2, and 5 s and for both the floating-point and INT8 model variants.

For the INT8 configuration, five acquisitions were analyzed separately. Peak power and incremental inference energy were calculated for each acquisition. The reported values are the means of the five results, and the ± values denote their standard deviations.

The utilization of program memory, data memory, stack, and working buffers was determined based on the map file generated during compilation and on measurement of the maximum stack usage. For each model version, the number of parameters, weight size, peak working memory demand, and the number of MAC operations per update were reported.

The data-rate analysis distinguished among the full ADC stream generated by the radar configuration, the reduced stream used by the phase-processing branch after chirp selection, and the output transmitted in the local-inference mode. Each complex ADC sample comprised a 16-bit I component and a 16-bit Q component, corresponding to 4 bytes per sample. Therefore, the full ADC data rate was calculated as 256 samples per chirp × 4 physical Rx channels × 48 chirps per frame × 20 frames/s × 4 bytes per complex sample, giving 3,932,160 B/s, or approximately 3.93 MB/s.

For the cardiorespiratory phase-processing branch, only one chirp corresponding to the fixed transmit antenna was selected from each frame, while samples from all four physical Rx channels were initially retained for range and receiver-channel selection. The data rate at this stage was therefore 256 × 4 × 1 × 20 × 4 = 81 920 B/s, or 81.92 kB/s. Thus, the value of approximately 81.9 kB/s represented the selected-chirp stream rather than the full ADC stream.

In the nominal local-inference configuration, the output packet contained 32 bytes and was transmitted once per second, corresponding to 32 B/s. The transmission reduction ratio was calculated as follows:

(13) $$R_{D}=\left(1-\frac{D_{local}}{D_{raw}}\right)\times 100\%,$$

Here *D_local_* denotes the data rate transmitted in the local-inference mode, and *D_raw_* denotes the selected reference data stream. The reduction ratio was calculated separately relative to the full ADC stream and to the reduced selected-chirp stream. The full ADC data rate was a theoretical rate generated by the stated acquisition configuration and does not imply that the complete stream was physically transmitted through the interface used in the target system.

Thermal stability was assessed during 60 min of continuous operation in the nominal configuration. A thermocouple was placed on the module surface above the AWR1843 device, while ambient temperature and humidity were recorded using a thermohygrometer. The steady-state temperature, its stabilization time, and any changes in inference time, power consumption, and HR/RR results across successive parts of the test were analyzed.

### 2.4. Statistical Analysis

The basic error metric was defined as the difference between the radar-derived result and the corresponding reference value:

(14) $$e_{i}={\hat{y}}_{i}-y_{i},$$

Here ${\hat{y}}_{i}$ denotes the HR or RR value estimated by the system, and *y_i_* denotes the corresponding reference value. Accuracy was evaluated using the mean absolute error (Equation (15)), the root mean square error (Equation (16)), and the systematic error (bias) (Equation (17)):

(15) $$MAE=\frac{1}{N}\sum_{i=1}^{N}\left|e_{i}\right|,$$

(16) $$RMSE=\sqrt{\frac{1}{N}\sum_{i=1}^{N}{e_{i}}^{2}},$$

(17) $$Bias=\frac{1}{N}\sum_{i=1}^{N}e_{i}.$$

All accuracy analyses were based on out-of-fold predictions. For each participant, the overall MAE was calculated by dividing the sum of absolute errors across all six scenarios by the total number of windows retained after quality control. The overall MAE reported in Table 3 was then calculated as the arithmetic mean of the 30 participant-specific MAEs, so that each participant contributed equally. For Table 4, MAE was calculated separately for each participant within each scenario and then averaged across participants. RMSE and bias were likewise calculated separately for each participant and then averaged across participants. The nominal window counts shown in Table 4 were not used as weights in these calculations.

The concordance correlation coefficient was calculated separately for HR and RR using the out-of-fold prediction–reference pairs. Its confidence interval was obtained using the participant-level cluster-bootstrap procedure described below. The accuracy metrics were additionally calculated separately for the resting condition, individual measurement distances, post-exercise recovery, and controlled motion.

To avoid treating overlapping windows as independent observations in the SQI analysis, Spearman’s rank correlation between SQI and absolute estimation error was calculated separately for each participant. The participant-specific correlation coefficients were summarized across participants, and a two-sided Wilcoxon signed-rank test was used to assess whether their median differed from zero. Consequently, the reported *p*-values were based on participant-level correlation coefficients rather than on the total number of overlapping observation windows.

The 95% confidence intervals were determined using a participant-level cluster bootstrap with 10,000 resamples. Participant identifiers, rather than individual observation windows, were sampled with replacement, and all retained windows belonging to a selected participant were included in each bootstrap sample. This procedure preserved the dependence between overlapping windows and repeated observations from the same participant. The 2.5th and 97.5th percentiles of the bootstrap distributions were reported as the lower and upper confidence limits.

Comparisons between model variants were performed on the basis of MAE values calculated separately for each participant. Paired comparisons were conducted using the Wilcoxon signed-rank test with Holm correction for multiple comparisons. The level of statistical significance was set at *p* < 0.05.

For each ablation, paired MAE differences were calculated separately for all 30 participants as the nominal-model MAE minus the ablation-model MAE, using the same retained output times within each pair. The 95% confidence interval for the mean paired difference was obtained by bootstrap resampling of the 30 participant pairs. In each of 10,000 resamples, participant pairs were sampled with replacement, and their mean difference was recalculated. The 2.5th and 97.5th percentiles of these means formed the confidence interval.

## 3. Results

### 3.1. HR and RR Estimation Accuracy

The final evaluation used predictions obtained for all 30 participants across five iterations of participant-independent cross-validation. In each iteration, the model was evaluated on data from six individuals who had not taken part in either its training or parameter selection. A comparison of the results obtained with the classical spectral estimator and the Tiny 1D CNN models is presented in Table 3.

The floating-point model achieved an HR MAE of 1.86 beats/min [95% CI: 1.65–2.10] and an RR MAE of 0.94 breaths/min [95% CI: 0.82–1.07]. Following INT8 quantization, these values increased to 2.09 beats/min [95% CI: 1.71–2.18] and 1.05 breaths/min [95% CI: 0.86–1.14], respectively. This corresponded to increases in MAE of 0.23 beats/min for HR and 0.11 breaths/min for RR.

Under the nominal short-window configuration, both neural-network variants achieved lower estimation errors than the implemented classical spectral baseline. Relative to this short-window spectral baseline, the INT8 model reduced the MAE by 45.3% for HR and 37.5% for RR.

Accuracy was evaluated using windows retained after reference-signal quality control. The same retained windows were used to compare the three estimation methods. Consecutive windows partially overlapped and therefore were not treated as statistically independent observations. The nominal numbers of candidate windows before quality control are reported in Table 4.

During post-exercise recovery, HR and RR estimates were obtained every 1 s. The MAEs in this scenario were 2.41 beats/min and 1.26 breaths/min, respectively. Tracking delay during the physiological transition was not separately quantified.

Repeated-measures Bland–Altman analysis of the INT8 model showed an HR bias of −0.10 beats/min. The lower limit of agreement was −5.47 beats/min (95% CI: −6.38 to −4.59), and the upper limit was 5.27 beats/min (95% CI: 4.41 to 6.15). For RR, the bias was 0.04 breaths/min. The lower limit of agreement was −2.68 breaths/min (95% CI: −3.08 to −2.19), and the upper limit was 2.76 breaths/min (95% CI: 2.35 to 3.24). The limits and their confidence intervals were estimated using the repeated-measures mixed-effects model and participant-level bootstrap described in Section 2.4. No clear increase in error magnitude was observed with increasing HR or RR. The agreement and Bland–Altman plots are presented in Figure 5.

SQI was negatively correlated with the absolute estimation error. Spearman’s rank correlation coefficient was −0.61 for HR and −0.66 for RR (*p* < 0.001). For windows with an SQI greater than 0.70, the MAE was 1.52 beats/min for HR and 0.76 breaths/min for RR. For an SQI below 0.40, these values increased to 4.18 beats/min and 2.16 breaths/min, respectively. These results confirmed that SQI could be used as a reference-free indicator of prediction reliability.

### 3.2. Results Under Different Measurement Conditions

The accuracy of the INT8 model depended on the measurement conditions. The lowest errors were obtained during resting measurements and at a distance of 1.0 m. Increasing the distance to 1.5 m, post-exercise recovery, and controlled motion resulted in reduced accuracy. Detailed results are presented in Table 4.

At distances of 0.5 and 1.0 m, the HR MAE did not exceed 1.62 beats/min, while the RR MAE did not exceed 0.78 breaths/min. Increasing the distance from 1.0 to 1.5 m increased the MAE to 2.16 beats/min for HR and 1.07 breaths/min for RR. This was accompanied by a decrease in the median SQI from 0.87 to 0.72.

During post-exercise recovery, the model tracked the gradual decreases in HR and RR; however, greater within-window variability resulted in increased estimation errors. In this scenario, the MAE was 2.41 beats/min for HR and 1.26 breaths/min for RR. The largest errors occurred during controlled motion. Compared with the resting condition, the HR MAE increased by 2.16 beats/min, while the RR MAE increased by 1.16 breaths/min.

The temporal characteristics of the estimates were determined by the causal observation windows and the update interval. The first HR estimate was available after 10 s of signal acquisition, whereas the first RR estimate was available after 30 s. Subsequent estimates were generated every 1 s and assigned to the end of the corresponding observation window. The computational delay of the nominal INT8 implementation was 2.8 ms for the complete update. Because the reference values and radar-derived estimates were calculated within the same temporally aligned windows, the reported accuracy analysis did not constitute a separate evaluation of tracking delay during rapid physiological transitions. A dedicated time-resolved assessment of transient response was not performed.

All scenarios included recordings from 30 participants. The window counts in Table 4 are nominal numbers of candidate windows calculated from the recording durations, the 10-s HR and 30-s RR observation windows, and the 1-s update interval. They do not represent the numbers retained after reference-signal quality control. Because consecutive windows partially overlapped, confidence intervals were calculated by resampling participants rather than individual windows, as described in Section 2.4.

Despite the reduction in accuracy during motion, the neural model continued to outperform the spectral estimator, for which the MAE values in the same scenario were 6.74 beats/min for HR and 3.12 breaths/min for RR. The error distributions across the measurement scenarios and their relationships with SQI are presented in Figure 6.

### 3.3. Model Selection and Ablation Analysis

The ablation analysis showed that increasing the HR window length from 6 to 10 s reduced the MAE from 2.38 to 1.86 beats/min. A further increase to 12 s provided only a minor improvement, reducing the MAE to 1.84 beats/min while increasing the latency of the first estimate. Therefore, a 10-s window was selected.

For RR, a 20-s window resulted in an MAE of 1.31 breaths/min. Increasing the window length to 30 s reduced the error to 0.94 breaths/min, whereas a 40-s window yielded an MAE of 0.90 breaths/min. Because the improvement between the 30- and 40-s windows was only 0.04 breaths/min, the 30-s window was selected for the final configuration.

To determine whether the use of branch-specific observation durations affected the reported performance, the nominal 10 s/30 s configuration was additionally compared with a shared-duration configuration using 30 s windows for both HR and RR. The contribution of SQI as a network input was evaluated separately using an otherwise identical model trained without the SQI value. The results of both experiments are presented in Table 5.

With a shared 30-s observation duration, the HR MAE was 1.88 beats/min, compared with 2.09 beats/min for the nominal multi-timescale configuration. The corresponding HR CCC values were 0.970 and 0.968. For RR, the MAE values were 0.98 and 1.05 breaths/min, respectively. The shared-duration configuration therefore had lower point estimates of MAE, but its first HR result became available after 30 s rather than 10 s. The nominal 10-s/30-s configuration was retained to provide an earlier first HR estimate; an accuracy advantage of the shorter HR window is not claimed.

Removing SQI as a model input produced mixed changes in the error metrics. The HR MAE decreased from 2.09 to 2.01 beats/min, whereas the RR MAE increased from 1.05 to 1.08 breaths/min. The corresponding CCC values decreased from 0.968 to 0.960 for HR and from 0.958 to 0.951 for RR. These results do not establish a consistent MAE advantage from supplying SQI to the regression branches. The relationship between SQI and estimation error is therefore discussed separately from its contribution as a model input.

Paired participant-level comparisons were calculated as the MAE of the nominal configuration minus the MAE of the corresponding ablation variant, using the same retained output times for each pair. For the shared-duration model, the mean paired differences were +0.22 beats/min for HR (95% CI: +0.01 to +0.38) and +0.06 breaths/min for RR (95% CI: −0.03 to +0.17). For the model without SQI, they were +0.09 beats/min for HR (95% CI: −0.04 to +0.22) and −0.04 breaths/min for RR (95% CI: −0.09 to +0.06). The 30-s shared-duration model had a lower HR MAE on matched output times, whereas the nominal configuration provided its first HR estimate 20 s earlier.

The nominal network contained approximately 4800 parameters and required 0.59 million MAC operations per joint HR and RR update. The small variant, containing 2100 parameters, achieved MAE values of 2.11 beats/min for HR and 1.11 breaths/min for RR. Increasing the model size to 14,600 parameters improved the respective MAE values to 1.80 beats/min and 0.91 breaths/min. The nominal architecture was therefore selected as a compromise among accuracy, model size, and computational cost.

### 3.4. Embedded Implementation Performance and the Accuracy–Energy Trade-Off

The results of the implementation on the AWR1843 are presented in Table 6. This device integrates a C674x DSP, an ARM Cortex-R4F processor, a hardware radar accelerator, and 2 MB of on-chip memory. Consequently, the model requires only a small fraction of the available memory resources.

The number of parameters, weight size, peak working memory, MAC count, and combined HR and RR inference time refer to the neural-network implementation. Peak working memory includes the activation and inference-workspace buffers required by both network branches but excludes the buffers used by the continuously operating radar-processing pipeline. Total estimation-update time includes input preparation, execution of both neural-network branches, and output-message generation. It excludes routine per-frame range-domain and phase-signal processing, which was performed separately at 20 Hz. The reported power values refer to the complete radar node rather than to the neural network alone. For the INT8 peak-power and incremental-energy entries, values are reported as the mean ± standard deviation across five acquisitions.

Quantization reduced the weight size by a factor of four and shortened the inference time from 4.6 to 1.9 ms. The incremental energy associated with a single model execution decreased by approximately 66.7%. Across the five INT8 acquisitions, the peak power was 2.88 ± 0.15 W and the incremental energy per inference was 0.9 ± 0.2 mJ (mean ± standard deviation). At the same time, quantization had little effect on the mean power consumption of the complete node because the radar front end, analog-to-digital conversion, and per-frame signal processing remained the dominant power consumers.

Increasing the update interval from 1 to 2 or 5 s did not produce a meaningful reduction in the mean power consumption of the complete node, which remained approximately 2.38 W in all three cases. Less frequent updates reduced the number of network executions but did not deactivate the radar transceiver. An update interval of 5 s reduced the frequency of available HR estimates during post-exercise recovery. A 1-s interval was therefore retained for the nominal configuration; the effect of update frequency on tracking delay was not quantified.

In the nominal local-inference mode, the device transmitted 32 B/s. The complete ADC stream generated by the stated configuration was calculated as 3,932,160 B/s (approximately 3.93 MB/s), based on 256 complex samples, four physical Rx channels, 48 chirps per frame, and 20 frames/s. The phase-processing branch used one selected chirp per frame across the four Rx channels, corresponding to a reduced input stream of 81,920 B/s (81.92 kB/s). Thus, the previously reported value of 81.9 kB/s represented the selected-chirp stream rather than the full ADC stream. Relative to the complete ADC stream, local inference reduced the transmitted data volume by approximately 99.9992%. Relative to the selected-chirp stream, the reduction was approximately 99.96%.

During the 60-min test, the module surface temperature increased from 25.6 °C to a steady-state value of approximately 46.2 °C at an ambient temperature of 22.4 °C. A near-steady state was reached after approximately 32 min. No interruptions in operation or evidence of frequency throttling were observed thereafter. The change in inference time between the beginning and end of the test did not exceed 0.7%, while the relative change in mean power was 0.5%.

Overall, the results showed that the INT8 variant maintained accuracy close to that of the FP32 model while reducing the weight size, execution time, and energy per inference. However, the complete-node analysis demonstrated that reducing the computational cost of the neural network alone did not produce a proportionally large reduction in total power consumption because most of the energy was consumed by the continuously active radar front end. The trade-off among accuracy, inference energy, and model size is presented in Figure 7, while the power and temperature profiles recorded during continuous operation are shown in Figure 8.

## 4. Discussion

The results confirm the feasibility of implementing a complete processing pipeline for contactless monitoring of heart and respiratory rates directly on the AWR1843 device. Radar signal processing, phase stabilization, signal quality assessment, and Tiny 1D CNN inference were all performed without using an external computer to calculate HR or RR. Therefore, the reported execution times, memory usage, and energy measurements characterize the actual embedded implementation rather than a model executed on a workstation.

The INT8 variant achieved an MAE of 2.09 beats/min for HR and 1.05 breaths/min for RR, with CCC values of 0.968 and 0.958, respectively. Bland–Altman analysis further showed that the mean differences between the radar-based and reference measurements were close to zero, indicating no consistent tendency to overestimate or underestimate either parameter. Under the nominal short-window configuration, the Tiny 1D CNN achieved lower errors than the implemented classical spectral baseline. Relative to this baseline, the INT8 model reduced the MAE by 45.3% for HR and 37.5% for RR. The lower errors may reflect the ability of the model to exploit the temporal structure of the signal and to reduce the influence of respiratory harmonics, local phase disturbances, and transient reductions in signal quality. However, part of the observed difference may also result from the limited frequency resolution of the spectral estimator. Therefore, these results should not be interpreted as demonstrating general superiority over all conventional frequency-domain or time-domain methods.

The achieved accuracy is comparable to results reported in the recent literature, although direct comparisons are limited by differences in radar devices, reference instruments, and measurement protocols. Yang et al. [8] obtained MAE values of 1.65 beats/min for HR and 0.79 breaths/min for RR using phase-continuity tracking and SQI. Khan et al. [17] reported corresponding values of 2.37 beats/min and 1.18 breaths/min using T-CNN models implemented on an external PYNQ-ZU platform. A distinguishing feature of the proposed system is that both radar signal processing and neural network inference are executed directly on the radar SoC without an additional computing platform.

The use of different temporal context lengths for HR and RR was justified by the different dynamics of the two signals. The 10-s HR window contained a sufficient number of cardiac cycles while retaining the ability to update the result frequently. For RR, a 30-s window was used because a shorter segment may contain only a few complete respiratory cycles, particularly during slow breathing. Downsampling the RR branch to 5 Hz reduced the buffer size and computational workload without discarding information relevant to the respiratory frequency range. The 1-s update interval determines how frequently successive results are generated rather than the time required to obtain the first estimate. The initial latency was 10 s for HR and 30 s for RR. However, the selected observation lengths also limit the frequency resolution of the spectral estimator. The nominal Fourier-bin spacing is inversely proportional to the observation duration. It is therefore 0.1 Hz for the 10-s HR window, corresponding to 6 beats/min, and approximately 0.033 Hz for the 30-s RR window, corresponding to approximately 2 breaths/min. Although three-point parabolic interpolation reduces the quantization error associated with selecting a single FFT bin, it does not eliminate the limitation resulting from the finite observation duration. A 60-s observation interval would reduce the nominal spacing to approximately 0.0167 Hz, corresponding to 1 beat/min or 1 breath/min, but would also increase the initial estimation latency and reduce the ability to follow rapid physiological changes.

The measurement conditions had a clear effect on system accuracy. The lowest errors were obtained during resting measurements and at a distance of approximately 1 m. The deterioration observed at greater distances can be attributed to reduced signal quality, whereas the increased errors immediately after exercise and during motion can be associated with rapid physiological changes and body displacements whose amplitudes exceed those induced by cardiac activity. The largest errors occurred during controlled motion, reaching 3.58 beats/min for HR and 1.84 breaths/min for RR. These results indicate that subject motion remains the primary factor limiting the accuracy of measurements obtained using a single radar.

The correlations between SQI and absolute estimation error were −0.61 for HR and −0.66 for RR, indicating that SQI provides information about the reliability of an estimate. The SQI-input ablation produced mixed results and did not demonstrate a consistent accuracy benefit from supplying SQI directly to the regression branches. Thus, the association between SQI and estimation error should be distinguished from its effect as a regression-model input; the ablation does not demonstrate a consistent MAE benefit from supplying SQI. In a practical system, low-SQI measurements could be flagged as uncertain, withheld, or used to trigger re-estimation. SQI may therefore remain useful as part of the reliability-assessment mechanism of an autonomous radar node.

INT8 quantization caused only a minor increase in error relative to FP32 while reducing the weight size from 19.2 to 4.8 kB, the inference time from 4.6 to 1.9 ms, and the incremental energy per network inference from 2.7 to 0.9 mJ. Consequently, the nominal INT8 variant provided the most favorable practical trade-off between accuracy and energy among the evaluated configurations. However, the mean power consumption of the complete node remained approximately 2.38 W because the active radar transceiver, power-supply circuitry, and fundamental radar signal processing accounted for most of the energy consumption. Reducing the cost of inference alone does not therefore necessarily result in a proportional reduction in the total power consumption of the device. This finding confirms the importance of evaluating embedded models at the whole-system level, in accordance with the energy-aware machine learning approach [1,2].

During the 60-min continuous-operation test, the module temperature stabilized at approximately 46.2 °C, while the variation in inference time did not exceed 0.7%. No interruptions or indications of performance throttling were observed. However, the measured value represented the module surface temperature rather than the junction temperature of the AWR1843 device. Local inference also reduced the transmitted output to HR, RR, SQI, and basic device-status information, eliminating the need for continuous transmission of raw radar samples.

The study was limited by the controlled laboratory environment, measurements involving one participant at a time, and the absence of participants with diagnosed cardiopulmonary disorders. The controlled disturbances also did not represent the complete range of movements encountered during daily activities. Furthermore, consecutive observation windows partially overlapped and therefore cannot be treated as fully independent samples. For this reason, the dataset was divided into training, validation, and test sets at the participant level.

The comparison of estimation methods was additionally limited to the implemented spectral baseline. Time-domain approaches based on peak detection or template matching [21,22] were not evaluated. Unlike a discrete spectral-peak estimator, these methods are not directly constrained by Fourier-bin spacing and could therefore provide a more demanding baseline, particularly for short observation intervals. In addition, the relative performance of the neural and spectral estimators was not evaluated using a common longer observation interval, such as 60 s. Consequently, the reported reductions in MAE are specific to the implemented 10-s HR and 30-s RR configurations. The absence of time-domain baselines and long-window comparisons limits the generality of the conclusions concerning the relative estimation accuracy. These comparisons should be included in future studies using a dedicated and consistently validated experimental protocol.

A formal time-resolved evaluation of tracking delay during rapid physiological changes was not included. Although successive estimates were updated every 1 s, their temporal response was inherently influenced by the causal 10-s HR and 30-s RR observation windows. Consequently, the presented results characterize estimation accuracy within temporally aligned observation windows but do not quantify the transient tracking delay of the system.

The energy measurements are specific to the radar configuration, power-supply arrangement, and update frequency used in this study. Five INT8 acquisitions were used to assess the variation between recorded inference events, as reported in Table 6. The complete experimental setup was not independently reassembled and measured in separate runs; therefore, the reported standard deviations characterize variation across acquisitions within the same setup, rather than between-run reproducibility. The 0.5% change in mean power during the 60-min continuous-operation test characterizes within-run stability. The uncertainty of the reported values additionally depends on the ±0.1% tolerance of the shunt resistor, the accuracy of the voltage measurements, and the oscilloscope acquisition settings. These results should therefore be interpreted as measurements obtained for the reported experimental setup and should not be extrapolated directly to other radar modules without separate measurements.

Future work should include long-term measurements outside the laboratory, different body positions, uncontrolled motion, the presence of multiple people, adaptive control of the inference frequency based on SQI, and a time-resolved evaluation of the system response during rapid physiological changes. A systematic comparison with time-domain peak-detection and template-matching methods, as well as an evaluation using longer observation intervals such as 60 s, should also be conducted.

## 5. Conclusions

This study presented an embedded system for contactless monitoring of heart and respiratory rates using a single 77 GHz FMCW radar, together with a device-level evaluation of its computational and energy costs. Radar signal processing, phase stabilization, signal quality assessment, and inference using a multiscale temporal Tiny 1D CNN were performed directly on the AWR1843 device. An external computer was not involved in determining HR or RR in the intended operating mode.

Using different temporal contexts for the two parameters accounted for the differences between cardiac and respiratory dynamics. The nominal INT8 configuration maintained high agreement with the reference measurements while preserving low estimation errors for both HR and RR.Under the nominal configuration using 10-s HR and 30-s RR windows, the INT8 model reduced the MAE by 45.3% and 37.5%, respectively, relative to the implemented short-window spectral baseline. These improvements are specific to the selected observation lengths and should not be interpreted as demonstrating general superiority over all frequency-domain or time-domain estimation methods.

Model quantization caused only a minor reduction in accuracy relative to the FP32 variant while decreasing the weight size from 19.2 to 4.8 kB, the inference time from 4.6 to 1.9 ms, and the energy associated with a single model update from 2.7 to 0.9 mJ. The nominal INT8 variant therefore provided the most favorable balance among accuracy, memory footprint, execution time, and inference energy within the evaluated configurations. However, the mean power consumption of the complete radar node remained approximately 2.38 W because the radar transceiver, power-supply circuitry, and fundamental signal-processing operations accounted for most of the total energy consumption. Therefore, the reduction in inference energy should not be interpreted as a proportional reduction in whole-node power consumption. During the 60-min continuous-operation test, no progressive increase in inference time or operational instability associated with module heating was observed.

The results demonstrate that complete HR and RR estimation using a lightweight neural model can be performed directly on a radar SoC without an additional computing platform or continuous transmission of raw data. SQI also enables measurements with limited reliability to be identified, which is important for autonomous monitoring systems. Future work should address uncontrolled operating conditions, different body positions, a larger and more diverse participant cohort, and adaptive control of the measurement and inference frequencies. A systematic comparison with time-domain peak-detection and template-matching methods, as well as an evaluation using common longer observation intervals such as 60 s, should also be conducted.

## Figures and Tables

**Figure 1:**
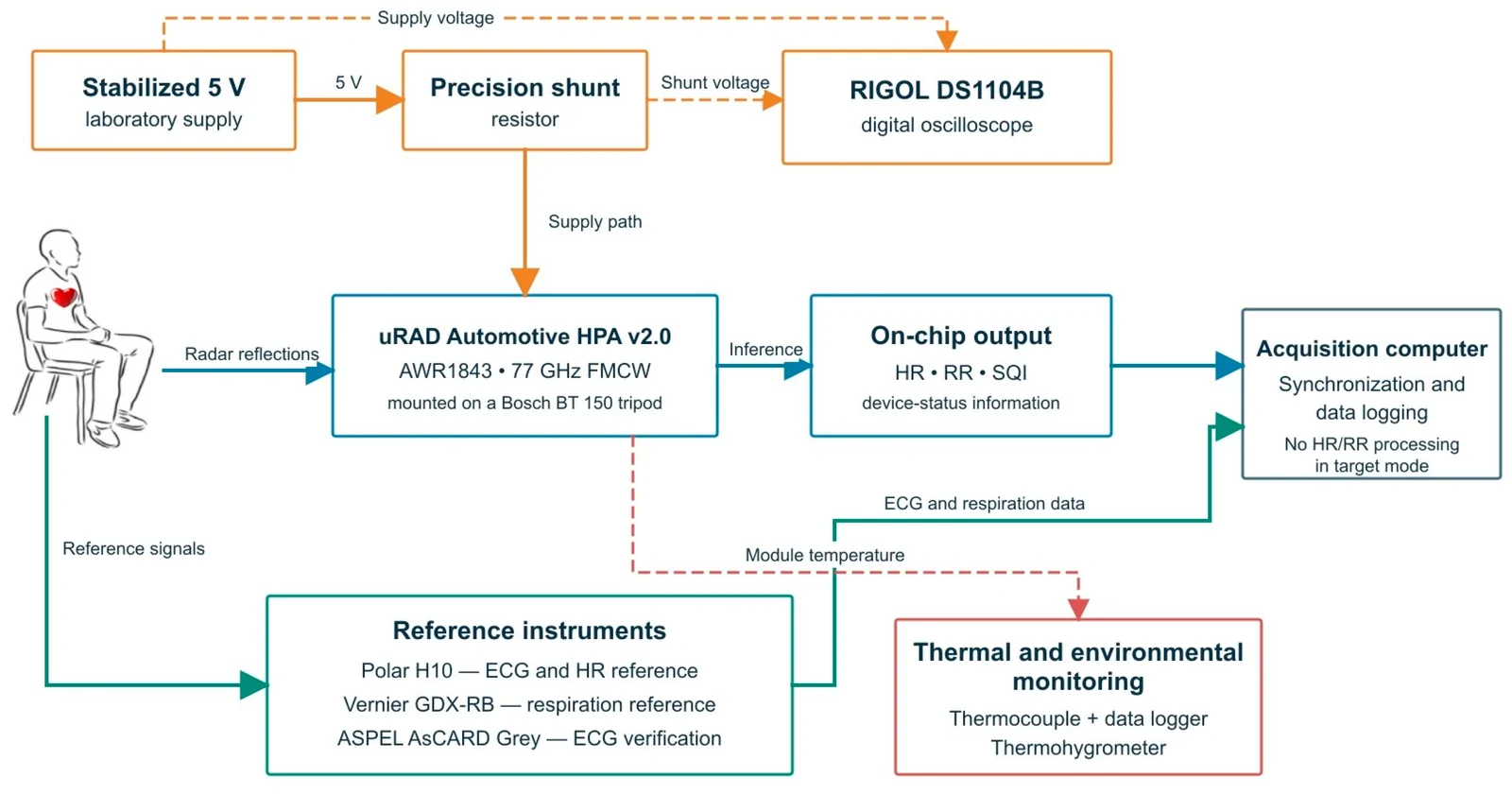
Block diagram of the measurement platform showing the radar and reference signal paths, on-chip HR and RR estimation, and power and thermal measurements.

**Figure 2:**
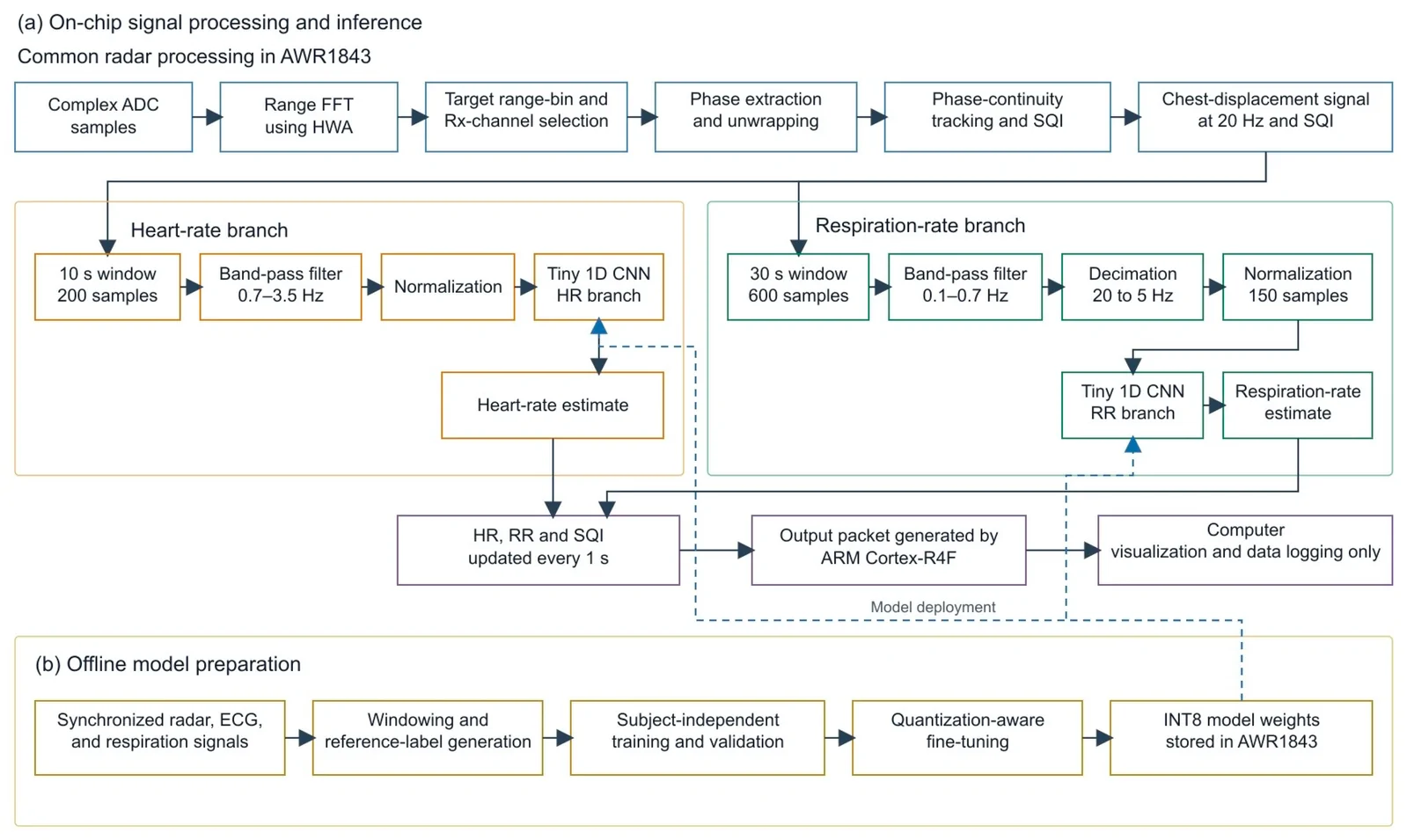
Signal processing and deployment workflow of the proposed embedded multi-timescale method for cardiorespiratory monitoring. The upper part shows signal processing and HR and RR estimation performed directly on the radar device. The lower part shows model preparation performed outside the radar and subsequent deployment of the model on the AWR1843 SoC.

**Figure 3:**
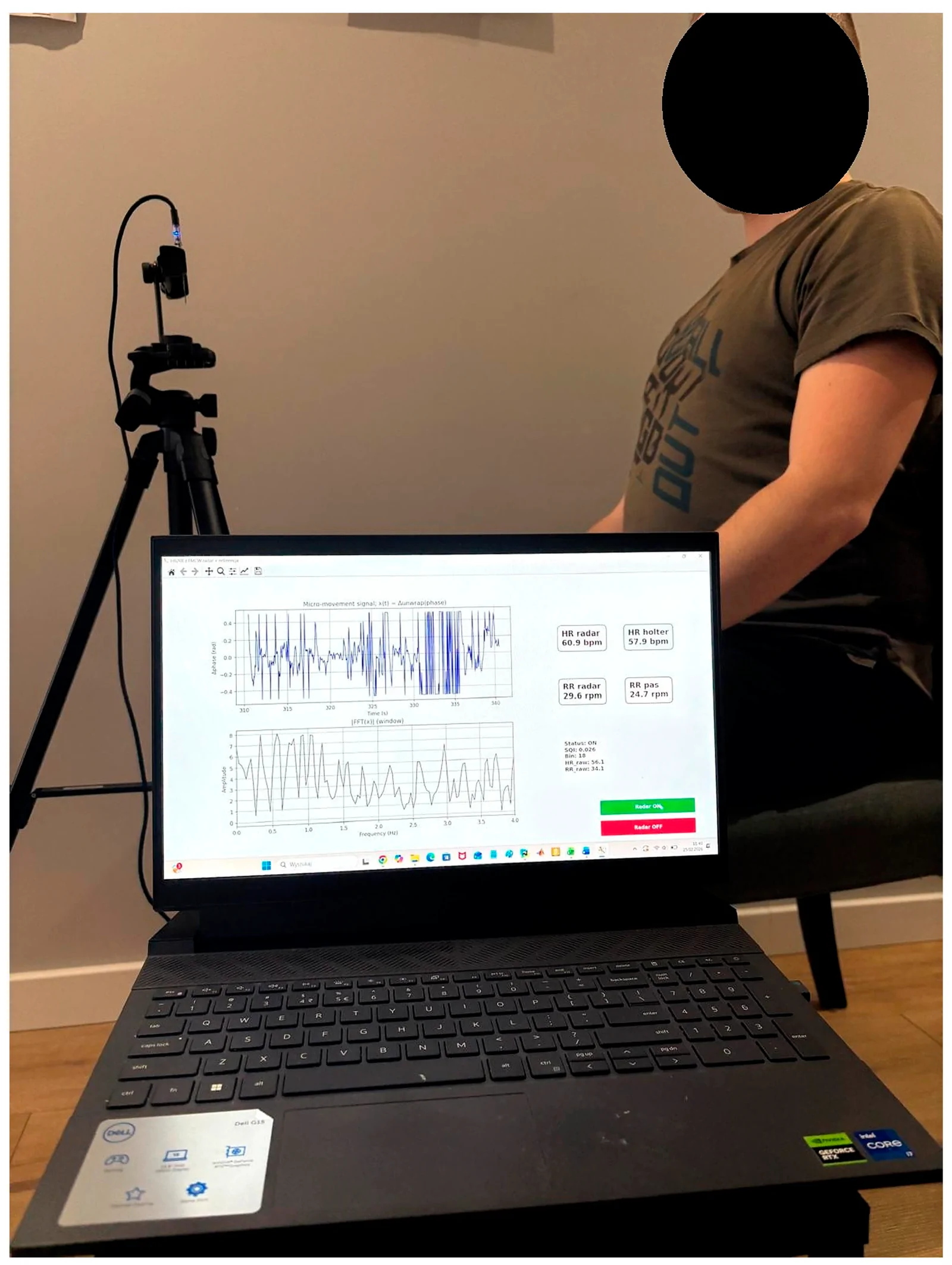
Experimental setup for contactless cardiorespiratory monitoring, showing a seated participant, a tripod-mounted 77 GHz FMCW radar positioned at chest level, and a computer used for synchronization and data recording.

**Figure 4:**
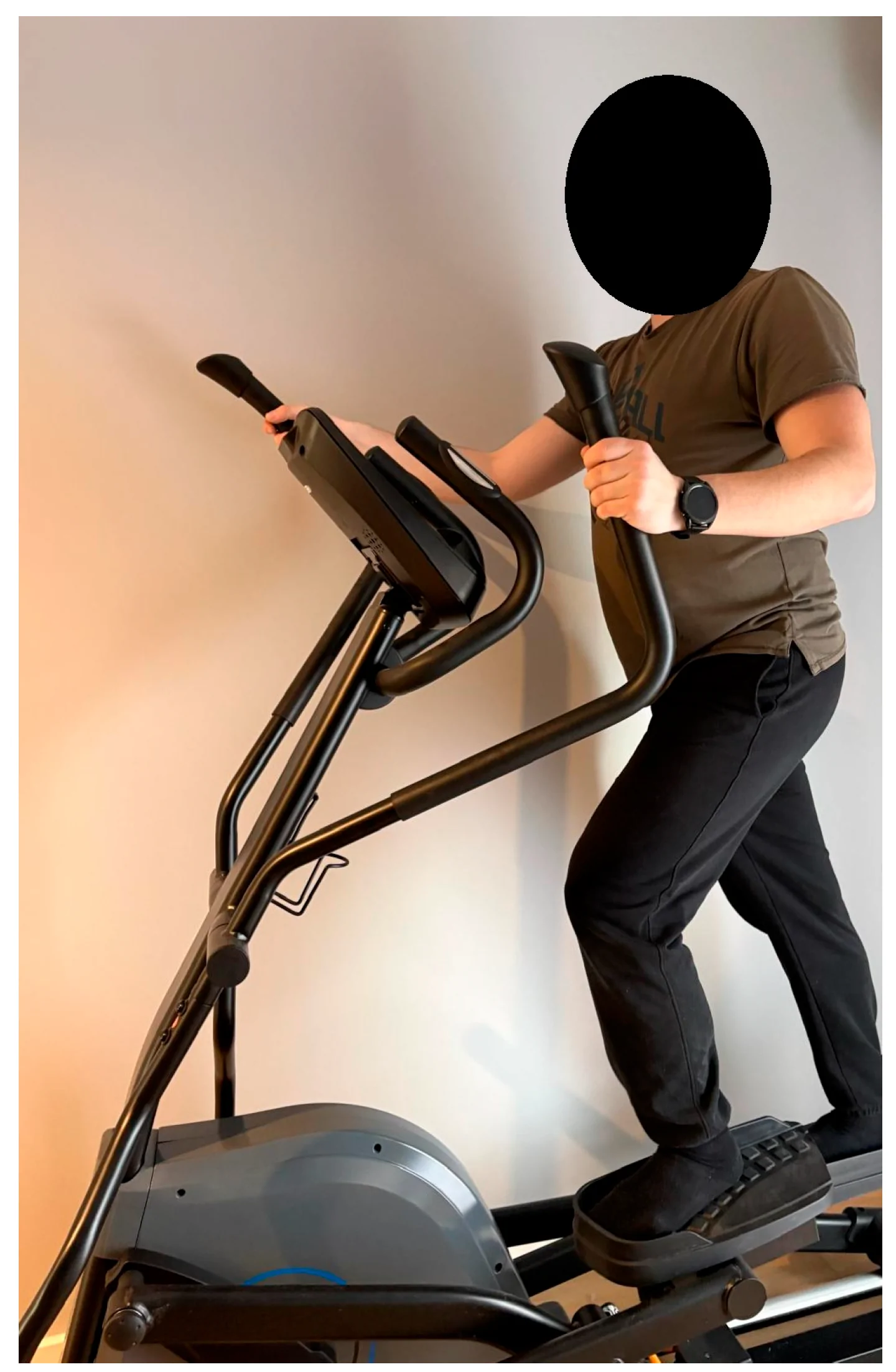
Moderate-intensity exercise performed on an elliptical trainer before recording the post-exercise recovery of HR and RR toward resting values.

**Figure 5:**
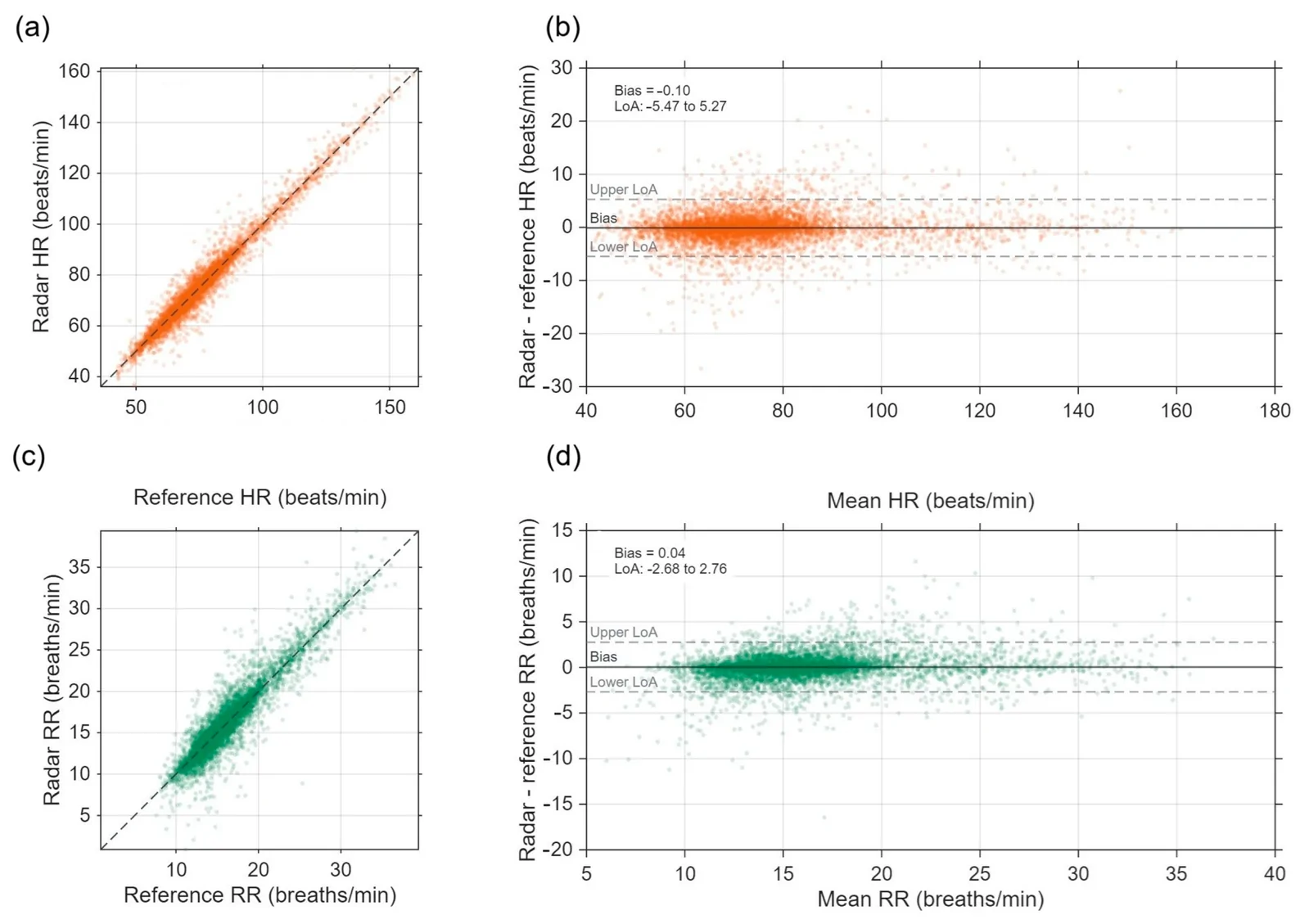
Agreement between the reference values and radar-based estimates: (**a**) regression plot for HR; (**b**) Bland–Altman analysis for HR; (**c**) regression plot for RR; and (**d**) Bland–Altman analysis for RR.

**Figure 6:**
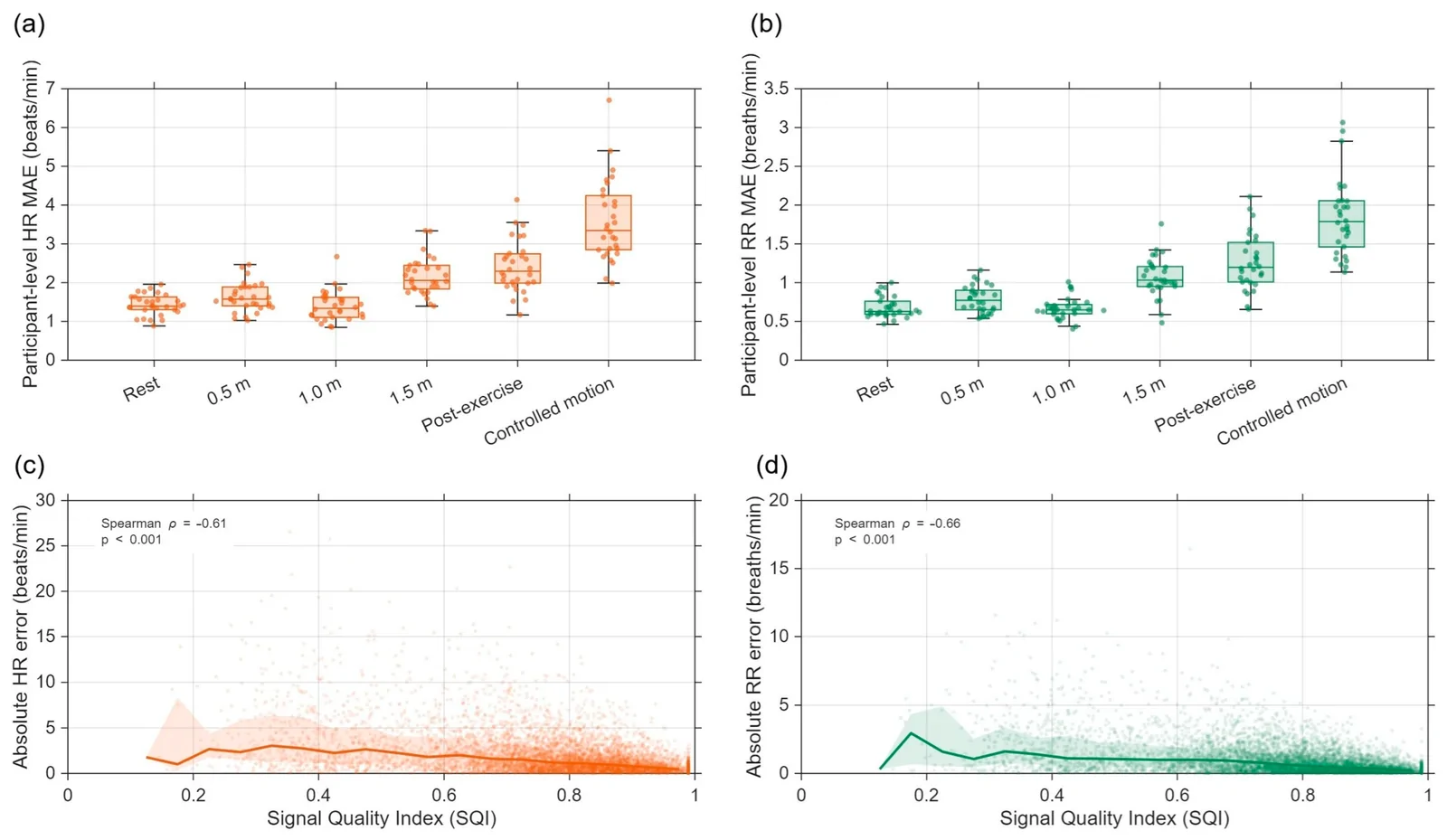
Effects of measurement conditions and signal quality on estimation accuracy: participant-level MAE for (**a**) HR and (**b**) RR across the experimental scenarios, and the relationship between SQI and absolute estimation error for (**c**) HR and (**d**) RR. The points in panels (**c**,**d**) represent individual analysis windows, while the solid lines and shaded regions indicate the median errors and interquartile ranges, respectively, calculated within SQI intervals.

**Figure 7:**
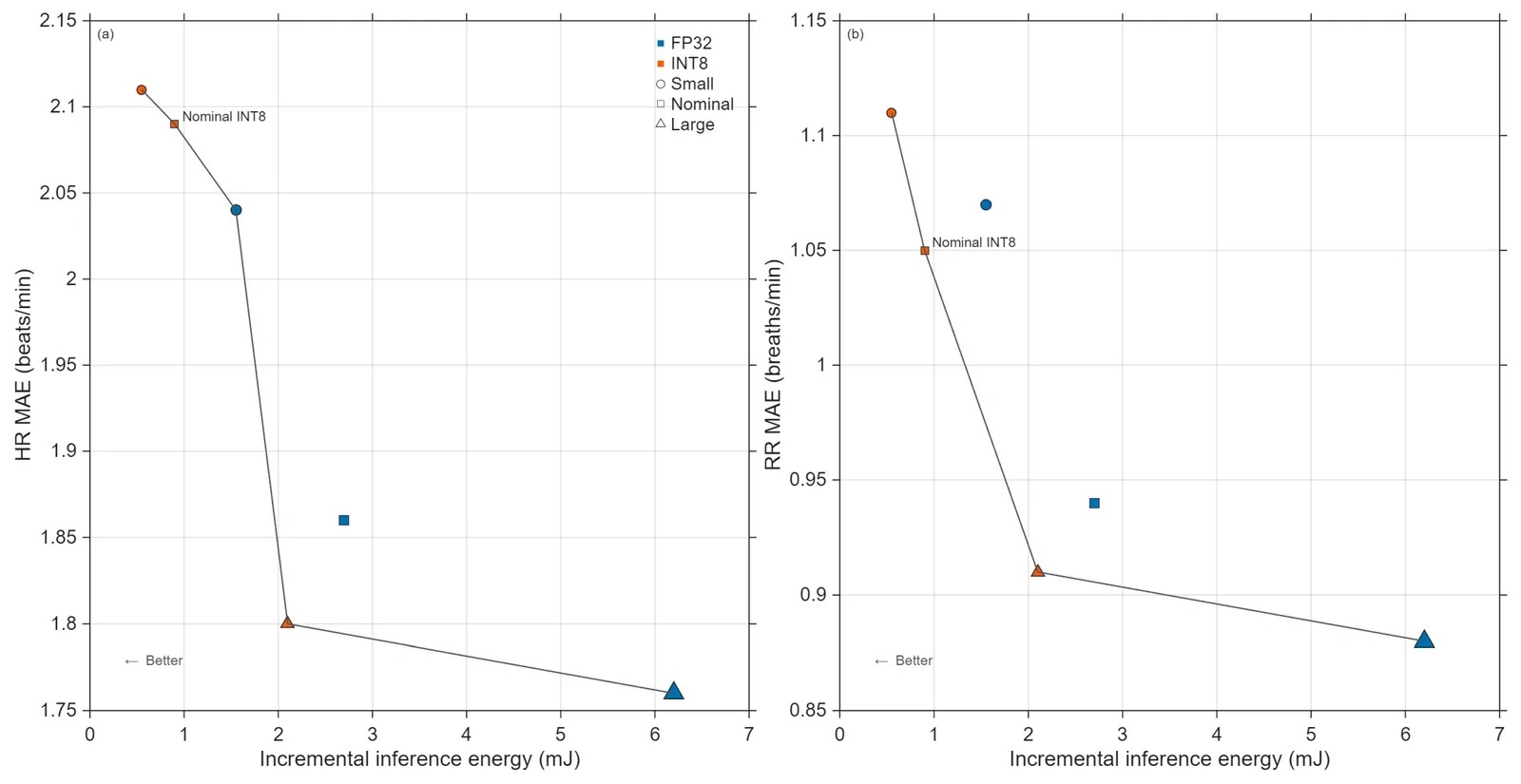
Accuracy–energy trade-off for the evaluated Tiny 1D CNN configurations: (**a**) HR estimation and (**b**) RR estimation. Marker color denotes numerical precision, marker shape denotes model size, and marker area is proportional to the memory occupied by the model weights. The gray line represents the Pareto frontier.

**Figure 8:**
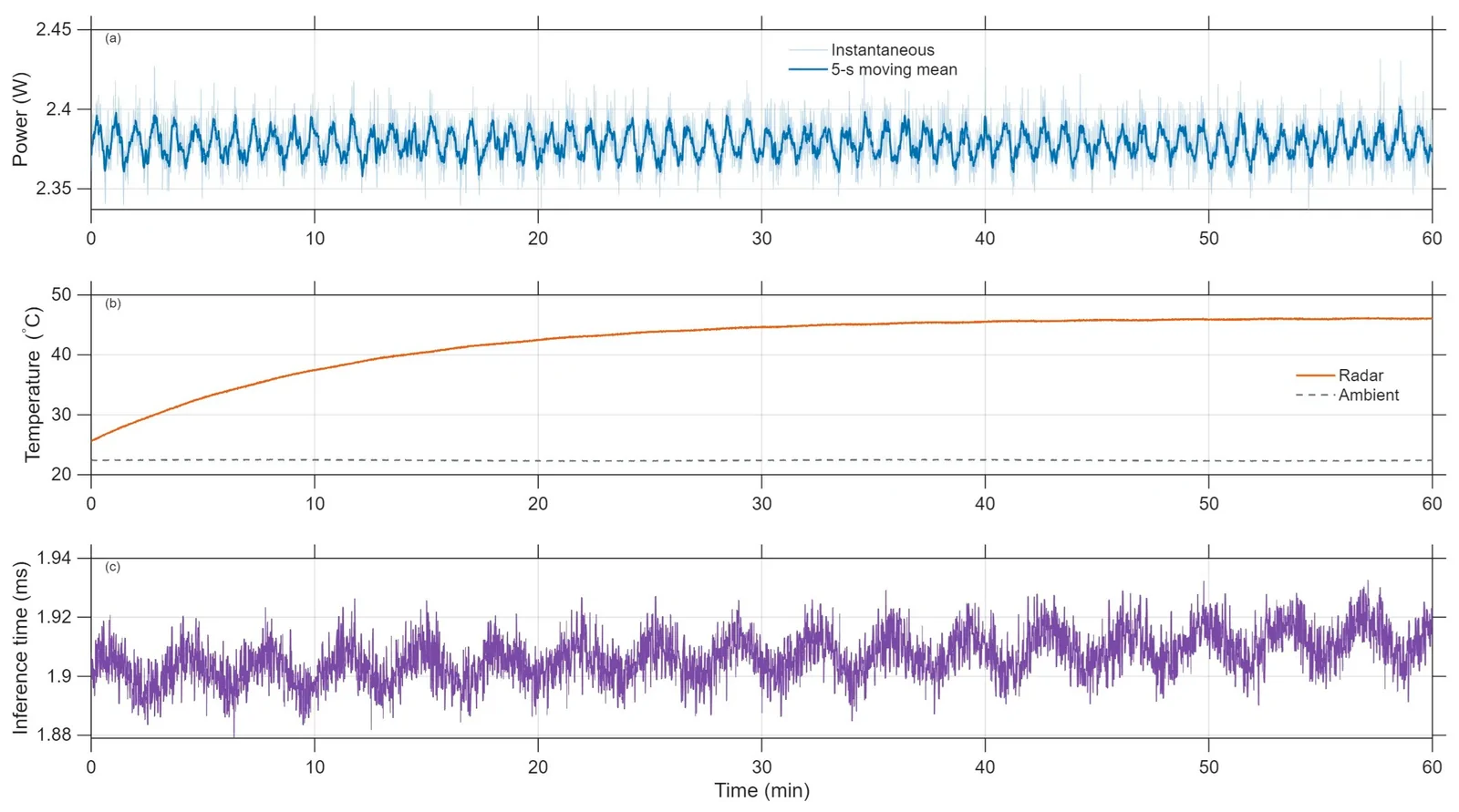
Results of the 60-min continuous-operation test: (**a**) power averaged over successive 1-s intervals and its 5-s moving average; (**b**) radar-module and ambient temperatures; and (**c**) Tiny 1D CNN inference time.

**Table 1:** Configuration of each branch of the multi-timescale Tiny 1D CNN.

Layer	Output Channels or Feature Dimension	Kernel Size	Dilation Rate	Activation
Conv1D	8	7	1	ReLU
Depthwise-separable Conv1D	8	5	1	ReLU
Depthwise-separable Conv1D	16	5	2	ReLU
Depthwise-separable Conv1D	24	5	4	ReLU
Depthwise-separable Conv1D	32	5	8	ReLU
Global average pooling	32	—	—	—
Concatenation with SQI	33	—	—	—
Fully connected layer	16	—	—	ReLU
Regression output	1	—	—	Linear

**Table 2:** Scenarios used for system validation.

Scenario	Participant Condition and Position	Recording Duration	Measurement Objective
Rest	Seated, 1.0 m from the radar	5 min	Assessment of accuracy under stable conditions
Distance variation	Seated at 0.5, 1.0, and 1.5 m, in an order varied across participants	3 min at each distance	Assessment of the effects of distance and signal level
Post-exercise recovery	After moderate exercise on an elliptical trainer	5 min	Assessment of changing HR and RR values
Controlled motion artifacts	Seated, 1.0 m from the radar	3 min per participant: three 30-s movement periods alternating with three 30-s periods of stillness	Assessment of the effects of body movements unrelated to cardiac and respiratory activity

**Table 3:** HR and RR estimation accuracy obtained under participant-independent validation. Values are presented as point estimates [95% participant-level cluster-bootstrap confidence intervals].

Method	Spectral Estimator	Tiny 1D CNN FP32	Tiny 1D CNN INT8
HR MAE (bpm)	3.82 [3.29, 4.37]	1.86 [1.65, 2.10]	2.09 [1.71, 2.18]
HR RMSE (bpm)	5.44 [4.79, 6.12]	2.65 [2.34, 3.00]	2.74 [2.42, 3.12]
HR Bias (bpm)	0.28 [−0.14, 0.68]	−0.08 [−0.25, 0.10]	−0.10 [−0.28, 0.08]
HR CCC	0.890 [0.852, 0.919]	0.970 [0.960, 0.978]	0.968 [0.957, 0.976]
RR MAE (breaths/min)	1.68 [1.43, 1.95]	0.94 [0.82, 1.07]	1.05 [0.86, 1.14]
RR RMSE (breaths/min)	2.36 [2.02, 2.73]	1.31 [1.13, 1.50]	1.39 [1.20, 1.60]
RR Bias (breaths/min)	0.12 [−0.04, 0.27]	0.03 [−0.05, 0.11]	0.04 [−0.05, 0.13]
RR CCC	0.880 [0.837, 0.914]	0.960 [0.944, 0.972]	0.958 [0.941, 0.970]

**Table 4:** Scenario-specific estimation accuracy and nominal candidate-window counts. Error metrics are presented as point estimates [95% participant-level cluster-bootstrap confidence intervals].

Scenario	Nominal Candidate Windows, n (HR/RR)	HR MAE (Beats/min)	HR RMSE (Beats/min)	RR MAE (Breaths/min)	RR RMSE (Breaths/min)	Median SQI
Rest, 1.0 m	8730/8130	1.42 [1.25, 1.60]	2.05 [1.79, 2.34]	0.68 [0.59, 0.78]	0.96 [0.83, 1.10]	0.86
Distance 0.5 m	4230/3630	1.62 [1.43, 1.83]	2.29 [2.00, 2.61]	0.78 [0.68, 0.89]	1.09 [0.94, 1.25]	0.82
Distance 1.0 m	4230/3630	1.39 [1.22, 1.58]	2.02 [1.76, 2.31]	0.67 [0.58, 0.77]	0.95 [0.82, 1.09]	0.87
Distance 1.5 m	4230/3630	2.16 [1.90, 2.45]	3.06 [2.67, 3.49]	1.07 [0.92, 1.23]	1.49 [1.29, 1.71]	0.72
Post-exercise recovery	8730/8130	2.41 [2.10, 2.75]	3.35 [2.91, 3.82]	1.26 [1.09, 1.45]	1.75 [1.51, 2.01]	0.70
Controlled motion	5130/4530	3.58 [3.06, 4.15]	4.89 [4.18, 5.68]	1.84 [1.56, 2.15]	2.49 [2.11, 2.91]	0.43

**Table 5:** Results of the observation-duration and SQI-input ablation analysis for the INT8 model.

Configuration	Nominal Multi-Timescale INT8 Model	Shared-Duration INT8 Model	INT8 Model Without SQI
HR window	10 s	30 s	10 s
RR window	30 s	30 s	30 s
SQI input	Yes	Yes	No
HR MAE	2.09	1.88	2.01
HR CCC	0.968	0.970	0.960
RR MAE	1.05	0.98	1.08
RR CCC	0.958	0.957	0.951

**Table 6:** Neural-network and complete-node implementation parameters for the FP32 and INT8 configurations on the AWR1843.

Parameter	FP32	INT8
Number of parameters	4 800	4 800
Neural-network weight size	19.2 kB	4.8 kB
Peak neural-network working memory	34.6 kB	31.2 kB
Neural-network MAC operations per update	0.59 million	0.59 million
Combined HR and RR neural-network inference time	4.6 ms	1.9 ms
Total estimation-update time	5.5 ms	2.8 ms
Complete-node peak power during inference	2.96 W	2.88 ± 0.15 W
Complete-node mean power	2.38 W	2.38 W
Incremental neural-network inference energy	2.7 mJ	0.9 ± 0.2 mJ

## Data Availability

The de-identified data supporting the findings of this study are available from the corresponding author upon reasonable request. The raw radar and physiological reference recordings are not publicly available because the participants did not consent to the public release of their individual data and to protect participant privacy.

## Abbreviations

The following abbreviations are used in this manuscript:

ADC	Analog-to-Digital Converter
AI	Artificial Intelligence
CCC	Concordance Correlation Coefficient
CNN	Convolutional Neural Network
DSP	Digital Signal Processor
ECG	Electrocardiography
FFT	Fast Fourier Transform
FMCW	Frequency-Modulated Continuous-Wave
FP32	32-Bit Floating-Point Format
HR	Heart Rate
INT8	8-Bit Integer Format
MAC	Multiply–Accumulate Operation
MAE	Mean Absolute Error
MIMO	Multiple-Input Multiple-Output
RR	Respiration Rate
SDK	Software Development Kit
SoC	System-on-Chip
SQI	Signal Quality Index

## References

[1] Różycki R., Solarska D.A., Waligóra G. (2025). Energy-aware machine learning models—A review of recent techniques and perspectives. Energies.

[2] Kang M., Park M. (2025). Power estimation and energy efficiency of AI accelerators on embedded systems. Energies.

[3] Sze V., Chen Y.-H., Yang T.-J., Emer J.S. (2017). Efficient processing of deep neural networks: A tutorial and survey. Proc. IEEE.

[4] Ostrysz M., Szczepaniak Z., Sondej T. (2026). Non-contact measurement of human vital signs in dynamic conditions using microwave techniques: A review. Sensors.

[5] Alizadeh M., Shaker G., de Almeida J.C.M., Morita P.P., Safavi-Naeini S. (2019). Remote monitoring of human vital signs using mm-wave FMCW radar. IEEE Access.

[6] Wang Y., Wang W., Zhou M., Ren A., Tian Z. (2020). Remote monitoring of human vital signs based on 77-GHz mm-wave FMCW radar. Sensors.

[7] Xiang M., Ren W., Li W., Xue Z., Jiang X. (2022). High-precision vital signs monitoring method using a FMCW millimeter-wave sensor. Sensors.

[8] Yang G., Metcalfe B.W., Watson R.J., Evans A.N. (2025). A noncontact cardiorespiratory monitoring system for drivers using millimeter wave radar with phase continuity tracking and signal quality index. IEEE Internet Things J..

[9] Yao S., Cong J., Li D., Deng Z. (2024). Noncontact vital sign monitoring with FMCW radar via maximum likelihood estimation. IEEE Internet Things J..

[10] Xiang Y., Guo J., Chen M., Wang Z., Han C. (2023). MAE-based self-supervised pretraining algorithm for heart rate estimation of radar signals. Sensors.

[11] Wang H., Du F., Zhu H., Zhang Z., Wang Y., Cao Q., Zhu X. (2023). HeRe: Heartbeat signal reconstruction for low-power millimeter-wave radar based on deep learning. IEEE Trans. Instrum. Meas..

[12] Li B., Li W., He Y., Zhang W., Fu H. (2024). RadarNet: Noncontact ECG signal measurement based on FMCW radar. IEEE Trans. Instrum. Meas..

[13] Kim D., Choi J., Yoon J., Cheon S., Kim B. (2024). HeartBeatNet: Enhancing fast and accurate heart rate estimation with FMCW radar and lightweight deep learning. IEEE Sens. Lett..

[14] Nocera A., Senigagliesi L., Raimondi M., Ciattaglia G., Gambi E. (2024). Machine Learning in RADAR-Based Physiological Signals Sensing: A Scoping Review of the Models, Datasets, and Metrics. IEEE Access.

[15] Jacob B., Kligys S., Chen B., Zhu M., Tang M., Howard A., Adam H., Kalenichenko D. Quantization and training of neural networks for efficient integer-arithmetic-only inference. Proceedings of the 2018 IEEE/CVF Conference on Computer Vision and Pattern Recognition.

[16] Mehrjouseresht P., El Hail R., Karsmakers P., Schreurs D.M.M.-P. (2024). Respiration and heart rate monitoring in smart homes: An angular-free approach with an FMCW radar. Sensors.

[17] Khan A., Wajid M., Srivastava A., Meena H.K., Farooq O. (2025). Contactless vital sign monitoring on edge devices with low-complexity T-CNN and mmWave FMCW radar. IEEE Sens. J..

[18] Khan A., Wajid M., Meena H.K., Srivastava A., Farooq O. MAVNet: Deep learning-based FMCW radar framework for motion-resilient vital sign monitoring on PYNQ SoC. Proceedings of the 2026 IEEE International Conference on Acoustics, Speech and Signal Processing (ICASSP 2026).

[19] Texas Instruments (2024). AWR1843 Single-Chip 77- to 79-GHz FMCW Radar Sensor.

[20] Anteral S.L. (2022). uRAD Automotive HPA v2.0: 77 GHz Radar, Velocity and 3D Positioning—Datasheet.

[21] Kim J.Y., Park J.H., Jang S.Y., Yang J.R. (2019). Peak detection algorithm for vital sign detection using Doppler radar sensors. Sensors.

[22] Yang Z., Yang H., Hu A., Zhuge X., Miao J. (2025). An adaptive template matching method for noncontact cardiac inter-beat interval detection using a 120 GHz FMCW radar. IEEE Trans. Instrum. Meas..

